# Biodegradable lipid nanoparticles for genome editing in the brain via intrathecal administration^☆^

**DOI:** 10.1016/j.mattod.2025.11.032

**Published:** 2026-01-23

**Authors:** Songtao Dong, Lauren Healy, Fanglin Gong, Yue Xu, Yunshu Cai, Nicholas C. Solek, Jingan Chen, Muye Zhou, Tyler Thomson, Margarita Savguira, Sijin Luozhong, Yanchao Zhang, Tingzhen He, Gen Li, Bowen Li

**Affiliations:** a Leslie Dan Faculty of Pharmacy, University of Toronto, Toronto, Ontario M5S 3M2, Canada; b Department of Chemistry, University of Toronto, Toronto, Ontario M5G 1L7, Canada; c Institute of Biomedical Engineering, University of Toronto, Toronto, Ontario M5S 3G9, Canada

**Keywords:** Ionizable lipid, Genome editing, Intrathecal administration, LNPs, Brain

## Abstract

Messenger RNA (mRNA)-based nonviral delivery of gene editors offers transformative potential for therapeutic genome editing in neurological diseases, but efficient and safe delivery to the brain remains a formidable challenge due to the restrictive blood–brain barrier. Intrathecal administration provides a clinically validated route to bypass this barrier, yet the design principles for biodegradable lipid nanoparticles (LNPs) optimized for central nervous system (CNS) delivery remain poorly defined. Here, we synthesized a 200-member combinatorial library of structurally diverse, biodegradable ionizable lipids using the Passerini three-component reaction. High-throughput *in vivo* screening identified P3B, a lead lipid incorporating degradable linkages and optimized ionizable head groups, which enables potent and well-tolerated intrathecal mRNA delivery. In Ai9 reporter mice, P3B-LNPs encapsulating Cas9 mRNA/sgRNA induced robust and widespread tdTomato expression in neurons and astrocytes across multiple brain regions, achieving substantially higher editing efficiency than the clinical benchmark DLin-MC3-DMA (MC3). In LumA reporter mice, P3B-LNPs mediated efficient adenine base editing, restoring luciferase expression throughout the brain with 14.8% on-target correction and minimal off-target activity. Compared with MC3, P3B-LNPs exhibited enhanced tolerability, with attenuated inflammatory responses and a safety profile supportive of repeated dosing. These findings establish P3B-LNPs as a potent, safe, and biodegradable platform for genome editing in the brain and underscore the power of combinatorial lipid chemistry and high-throughput *in vivo* screening to accelerate the development of next-generation LNPs for CNS-targeted mRNA therapeutics.

## Introduction

Messenger RNA (mRNA)-based delivery of gene editors has emerged as a transformative approach for precise genome editing, enabling the transient expression of programmable nucleases such as Cas9 and adenine or cytosine base editors [1,2]. This strategy builds on the clinical success of the Pfizer-BioNTech and Moderna COVID-19 vaccines, which demonstrated the safety, scalability, and versatility of LNP-mediated mRNA delivery [3]. Since then, mRNA-based strategies have rapidly expanded to diverse applications, including cancer immunotherapy [4–6], protein replacement [7–9], and genome editing [10,11]. Among these, gene editing in the brain represents a particularly promising but challenging frontier. Many neurological diseases, including neurodegenerative disorders, developmental syndromes, and brain tumours, are driven by well-characterized genetic mutations and could be potentially cured by targeted gene correction or modulation [12,13]. However, the large size and structural complexity of gene editors pose substantial barriers for delivery: Cas9 mRNA exceeds 4.5 kb, and base editors can reach 5.5 kb, making them significantly larger and more difficult to encapsulate and deliver than conventional therapeutic mRNAs. These size constraints, combined with the restrictive blood–-brain barrier, severely limit the efficiency and distribution of gene editors in the central nervous system. Collectively, many neurological disorders contribute substantially to global morbidity and mortality, and remain incurable despite significant advances in symptomatic treatment [14]. This therapeutic potential has fueled growing efforts delivery platforms capable of safely and efficiently targeting gene editors to the brain[15]. To date, however, efficient nonviral delivery of large mRNA-based gene editors, particularly base editors, to the adult brain has not been achieved, representing a major bottleneck in CNS genome editing.

Lipid nanoparticles (LNPs) have emerged as the most clinically advanced non-viral vectors for mRNA-based gene editor delivery, offering a well-established safety profile, scalability, and proven clinical utility [16]. Their modular design enables formulation tuning for diverse tissue targets, exemplified by the landmark clinical success of Intellia’s LNP-based CRISPR therapy for liver-directed genome editing [10,17,18]. LNPs have also been engineered to enable genome editing in spleen, and lungs [19–21]. Despite these advances, there remains a critical unmet need for delivery systems capable of achieving potent, safe, and widespread mRNA-based gene editor delivery to the brain.

The brain is protected by the blood–brain barrier (BBB), a highly selective endothelial interface that tightly regulates the passage of molecules from the bloodstream into the central nervous system (CNS) [13]. The brain’s complex anatomy further hinders access to specific regions and cell types [22]. Consequently, conventional systemic delivery strategies often fail to achieve sufficient expression in target brain tissues [23,24]. To address this, previous studies have explored both -strategies to cross the BBB and alternative administration routes [25]. For example, Wang et al. generated a library of BBB-crossing LNPs by incorporating brain-targeting small molecules into ionizable lipids, achieving measurable but limited expression in the brain [26]. However, BBB-crossing formulations are limited by low efficiency, cortical surface accumulation, and poor penetration of deeper brain regions [23,26,27]. Localized delivery methods, including intracerebral (IC) for direct parenchymal injection and intracerebroventricular (ICV) for cerebrospinal fluid (CSF)-mediated delivery, can bypass the BBB entirely, allowing accumulation of mRNA in targeted brain regions [22,28,29]. Although effective, these routes are inherently invasive, posing a significant translational barrier [13,30].

Intrathecal (IT) administration represents an alternative route that addresses these limitations. This route allows delivery directly into the CSF, which is produced in the brain ventricles and circulates through the subarachnoid space surrounding the brain and spinal cord [13,27,31]. Through exchange with the interstitial fluid of the CNS parenchyma, the CSF enables distribution of therapeutics throughout the entire CNS, while limiting systemic exposure [31,32]. IT is minimally invasive, supports repeated dosing, and is already employed clinically for anesthetics, chemotherapeutics, and antisense oligonucleotides [33–36]. However, efficient mRNA delivery to the brain by IT-administered LNPs is not yet established and poses a key challenge for the field. A recent study by Xue et al. presented rationally designed “TD” ionizable lipids, which achieved broad neuronal and astrocytic expression in mice following a single IT dose [27]. These findings demonstrate feasibility but highlight that rational design approaches, which explore only a narrow region of chemical space, have had limited success due to the complex and poorly understood factors governing CNS delivery. To build on this progress, a high-throughput synthesis approach that expands the diversity of ionizable lipids is warranted to identify novel structures with enhanced CNS delivery efficiency.

Here, we generated a 200-member library of biodegradable ionizable lipids using the Passerini three-component reaction (P-3CR)[37], a chemistry previously applied to combinatorial lipid synthesis but not to mRNA-based genome editing or CNS-directed gene delivery. From this library, we identified P3B, a lead lipid that mediated efficient, widespread mRNA delivery throughout the brain following IT administration. When formulated with mRNA encoding CRISPR-Cas9, P3B-LNPs achieved robust neuronal and astrocytic transfection and efficient on-target gene editing *in vivo*, surpassing the performance of the clinically benchmarked lipid DLin-MC3-DMA (MC3). Importnatly, P3B-LNPs also mediated on-target adenine base editing *in vivo*, achieving effective single-nucleotide correction in the adult mouse brain. To our knowledge, this represents the first demonstration of nonviral base editing in the adult CNS using LNPs. Furthermore, P3B-LNPs maintained a favourable safety profile with minimal systemic exposure or inflammatory activation. Collectively, these findings establish P3B as a distinct class of biodegradable ionizable lipids that enable efficient, safe and translatable CNS genome editing via intrathecal mRNA delivery.

## Results and Discussion

### Combinatorial ionizable lipid synthesis by P-3CR

**Fig. 1.**
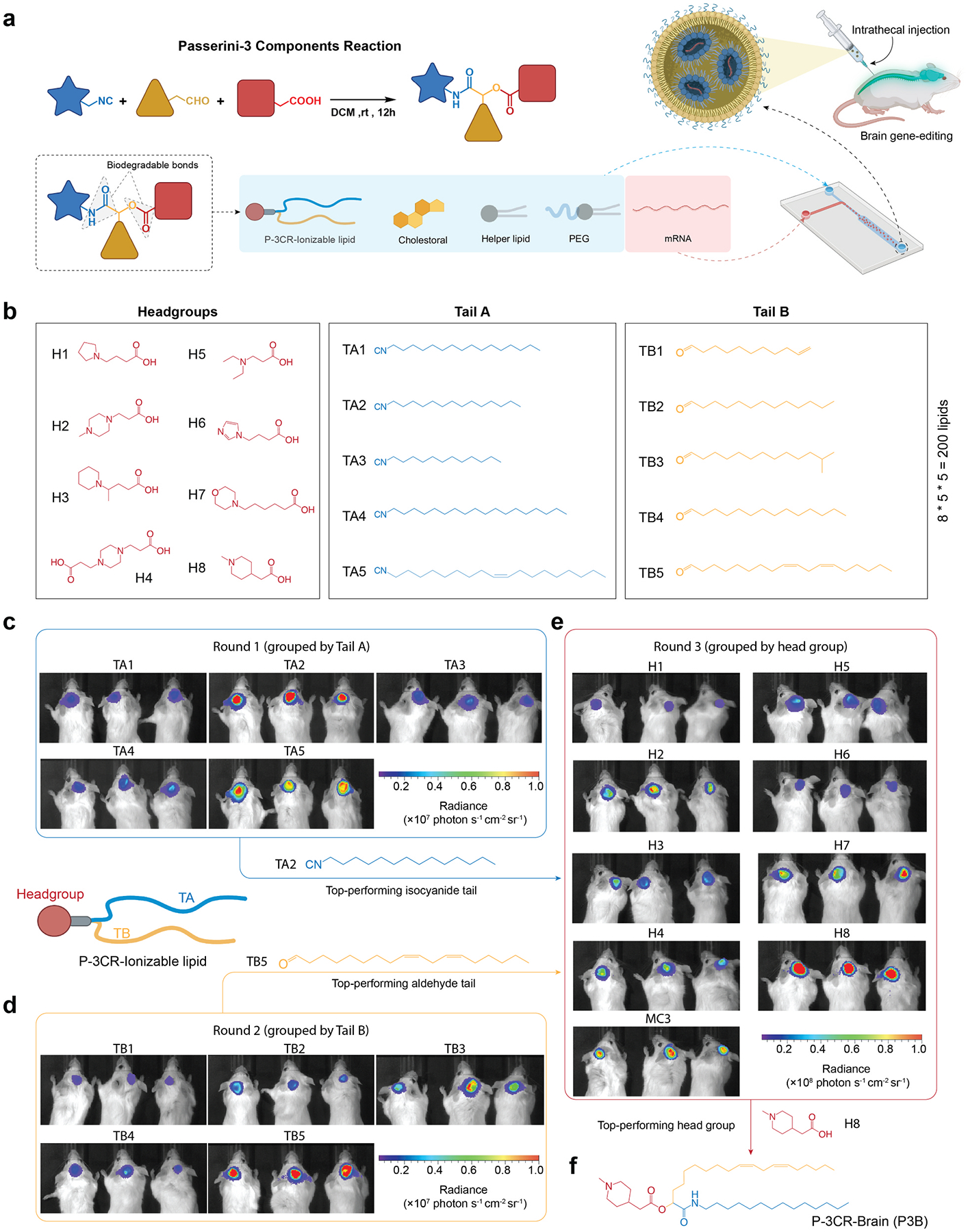
Design and synthesis of ionizable lipids by Passerini reaction. **(a)** Overview of the P-3CR chemistry for one-step ionizable lipid synthesis, combinatorial library generation via high-throughput screening (HTS), and formulation into mRNA-LNPs for IT delivery to the brain. **(b)** Chemical structures of the three constituents used to generate the 200-candidate library including carboxylic acid (headgroup), isocyanide (tail A), and aldehyde (tail B). **(c-e)** Round-wise *in vivo* screening of the library for firefly luciferase mRNA (mFluc) delivery after IT administration (0.05 mg/kg mRNA/mouse). Round 1 identified the top-performing Tail A, Round 2 identified the top-performing Tail B, and Round 3 identified the top-performing headgroup. n = 3 biological replicates. **(f)** Chemical structure of lead lipid P3B, identified by combining TA2, TB5, and H8.

To generate our ionizable lipid library, we employed the Passerini three-component reaction (P-3CR), a one-pot condensation of carboxylic acids (tertiary amine–containing headgroups), isocyanides (Tail A), and aldehydes (Tail B) (Fig. 1a). This approach enables the combinatorial creation of ionizable lipids under mild catalyst-free conditions, achieving high yield, diversity, and biodegradability. Compared with conventional multistep synthesis or focused rational design, P-3CR accelerates both the speed and diversity of lipid discovery [37,38]. In addition, it intrinsically yields an α-acyloxy amide scaffold containing a hydrolyzable ester bond, which confers biodegradability while eliminating the need for separate esterification steps. This is particularly important for brain-targeted materials, as parenchymal tissue is highly vulnerable to damage and degeneration arising from prolonged inflammatory states in the CNS [39,40].

Using P-3CR, we synthesized a combinatorial library of 200 lipids for high-throughput IT screening (Fig. 1a–b). The library included eight tertiary amine-containing carboxylic acid headgroups (H1-H8), five isocyanide tails (TA1-TA5), and five aldehyde tails (TB1-TB5), varying in tail chain length, structure and saturation (Fig. S1–S11). Conveniently, previous work has established that the P-3CR generates lipids at an acceptable purity for high-throughput screening [37]. The resulting ionizable lipids were formulated into LNPs using a classical four-composition formulation ratio, containing 1,2-distearoyl-*sn*-glycero-3-phosphocholine (DSPC) as a helper lipid, cholesterol, 1,2-dimyristoyl-*sn*-glycerol-3-phosphoethanolamine-N-[methoxy-(polyethyleneglycol)-2000] (C14-PEG), and the synthesized ionizable lipids from the P-3CR library.

### P-3CR LNP transfection efficiency for brain delivery

To identify lead candidates for IT mRNA delivery, the 200-member P-3CR lipid library was formulated into LNPs encapsulating firefly luciferase mRNA (mFluc) and screened *in vivo* following a single IT injection in mice (Fig. c–d). Direct *in vivo* screening was employed as *in vitro* systems fail to replicate the unique CSF environment or reflect the biodistribution of LNPs following IT administration. But, to efficiently evaluate all 200 LNPs *in vivo*, a batch-based screening approach was adopted to determine the influence of each component (Headgroup, Tail A and Tail B) on CNS delivery efficiency while reducing animal use, time, and cost [37,41]. In Round 1, formulations were pooled by isocyanide tail (Tail A) and administered by IT injection (Fig. 1c, Fig. S12). Two variants outperformed the others: TA2, a shorter linear chain, and TA5, a longer chain containing an unsaturation. TA2 achieved the highest mFluc expression, notably outperforming longer, fully saturated tails. In Round 2, lipids containing TA2 were advanced and pooled by aldehyde tail (Tail B) for further IT evaluation (Fig. 1d, Fig. S13). Here, TB3, which introduces a bulky *tert*-butyl group, and TB5, which carries two unsaturated bonds, outperformed the other Tail B variants. TB5 achieved the highest mFluc expression, although the structural differences between TB3 and TB5 indicated no consistent trend across the set. In Round 3, with TA2 and TB5 fixed, LNPs were evaluated individually by headgroup structure and administered by IT injection (Fig. 1e, Fig. S14). H8 consistently yielded the strongest luminescence across the CNS, while H2 and H7 showed intermediate activity. These findings are consistent with prior work demonstrating that subtle differences headgroup chemistry can strongly influence potency [19,42,43]. Our iterative strategy enabled rapid down-selection to a single lead structure, H8TA2TB5, designated P-3CR-Brain (P3B) (Fig. 1f), for further optimization and benchmarking.

### Formulation optimization of P3B

**Fig. 2.**
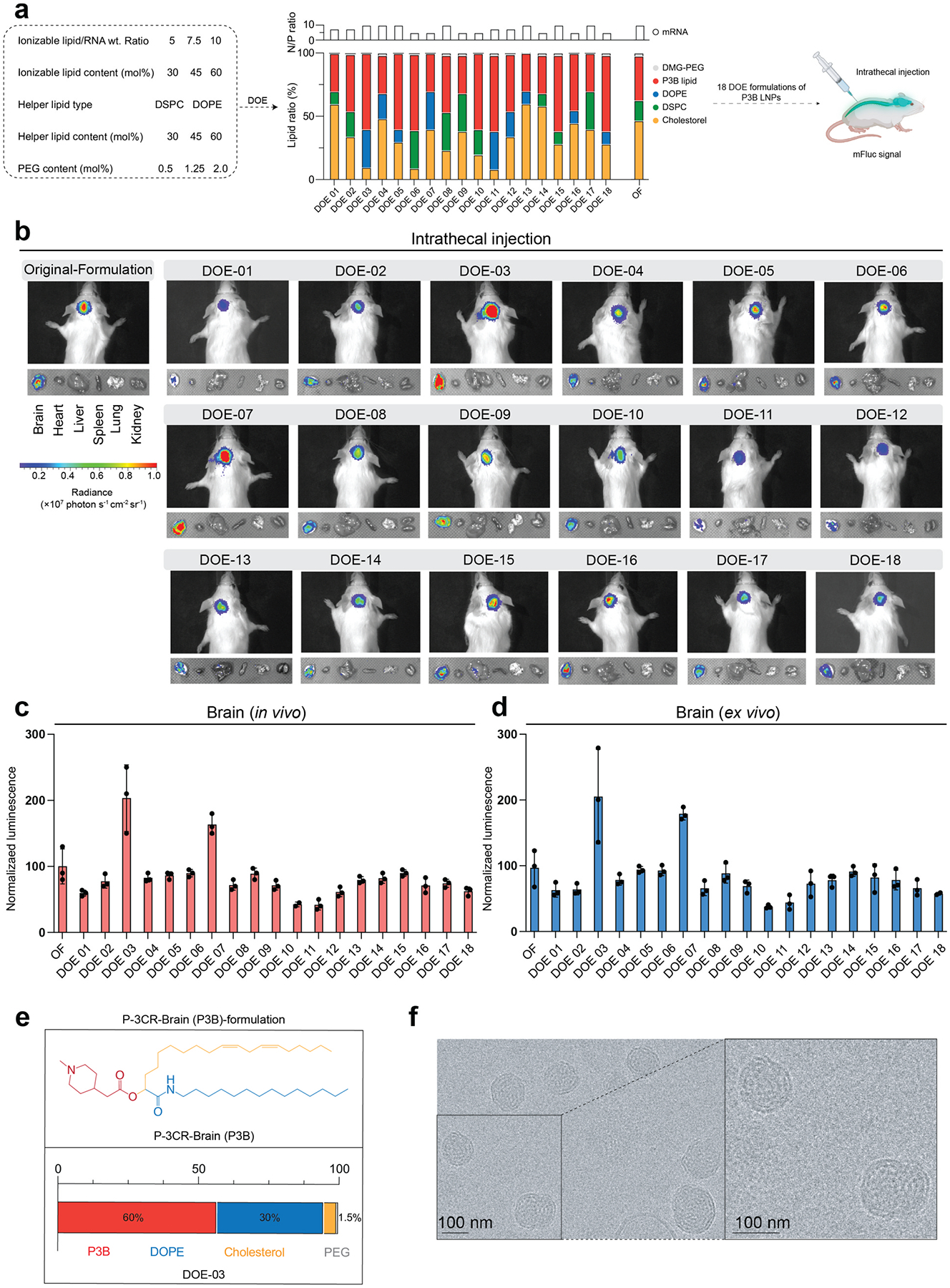
P3B LNP formulation optimization. **(a)** Library of 18 DOE formulations with varied parameters, encapsulating mFluc mRNA for intrathecal delivery, alongside the original formulation (OF). **(b)** Representative whole-animal and *ex vivo* organ bioluminescence imaging following intrathecal administration of DOE mFluc-LNPs (0.05 mg/kg mRNA/mouse). Representative images selected from n = 3 biological replicates per formulation, imaged under identical acquisition settings. **(c-d)** Quantification of Fluc protein expression in the brain *in vivo* and *ex vivo*. Data are presented as mean ± s.d.; n = 3 mice per group (biological replicates). **(e)** Composition of the optimised P3B formulation (P3B-B). **(f)** Cryo-EM imaging of P3B LNPs.

To optimize transfection potency of our lead ionizable lipid P3B, we implemented a Design of Experiment (DOE) strategy that systematically evaluated five formulation parameters: (1) ionizable lipid-to-mRNA mass ratio, (2) ionizable lipid molar fraction, (3) phospholipid type, (4) phospholipid molar fraction, and (5) PEG-lipid content (Fig. 2a) [38,44]. The levels tested for these variables are displayed in Fig. 2a, and detailed formulations are listed in the supporting information (SI) (SI Table 1). Eighteen formulations were prepared containing mFluc, and the transfection efficiency was evaluated *in vivo* following IT administration. Bioluminescence imaging showed marked differences in signal intensity between formulations (Fig. 2b). Importantly, expression was consistently localized to the brain with negligible off-target activity in peripheral organs, underscoring one of the key advantages of IT administration for achieving brain-targeted delivery [31,32]. Both DOE-3 and DOE-7 outperformed the original formulation, with quantification confirming DOE-3 exhibited the greatest potency. This result was consistent in both *in vivo* live imaging and *ex vivo* analysis of isolated brains (Fig. 2c–d, Fig. S15). Compared with DOE-7 (30 mol% P3B), DOE-3 contained a higher ionizable lipid fraction (60 mol%). In the context of IT delivery, this suggests that efficacy was improved by higher ionizable lipid content, consistent with the central role of ionizable lipids in stabilizing mRNA cargo and facilitating endosomal escape [45]. The optimal formulation tailored for P3B was defined by the following parameters: 10:1 ionizable lipid/mRNA mass ratio with 60 % P3B lipid, 30 % DOPE, 0.5 % PEGylated lipid molar composition. This optimized P3B formulation was used in all subsequent experiments. Cryo-EM imaging of optimized P3B LNPs showed spherical nanoparticles with uniform morphology and diameters of ~ 100 nm, without obvious aggregation or irregular structures (Fig. 2f). LNPs characterizations including mean hydrodynamic diameter (110 nm) and PDI (0.108) are consistent with well-formed LNPs and support their suitability for *in vivo* delivery [46,47]. The measured pKa of P3B LNPs was 6.51 (Fig. S16). Furthermore, long-term stability studies showed that P3B LNPs remained stable in PBS for up to 28 days at 4 °C (Fig. S17).

### Efficacy of P3B-LNPs for brain-specific gene editing

**Fig. 3.**
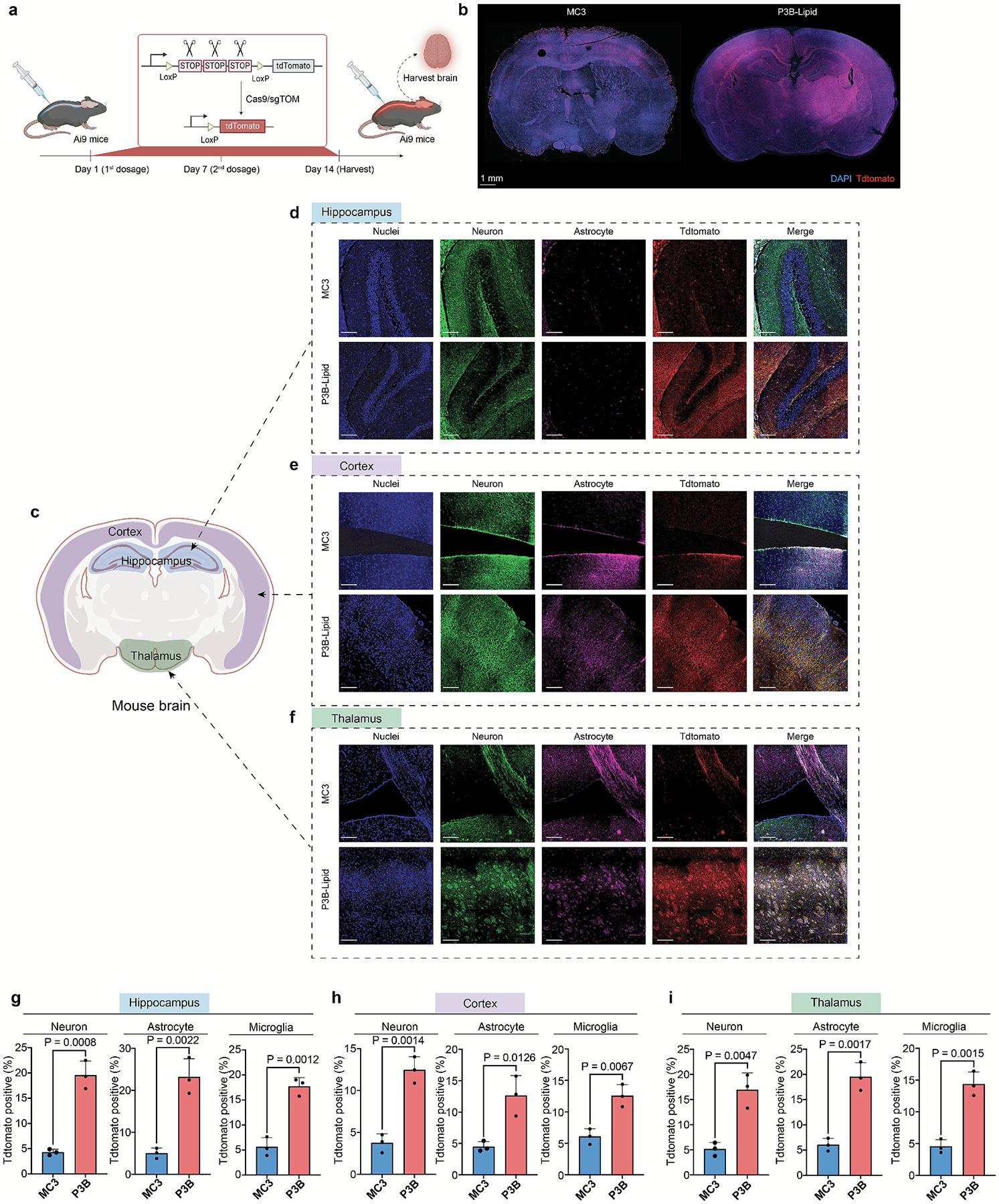
P3B-LNPs for Cas9 mRNA delivery in the Ai9 mouse model. **(a)** Diagram depicting Cas9 mRNA/sgTOM delivery removing the LoxP-flanked stop cassette to activate tdTomato expression in Ai9 mice. **(b)** Representative whole-brain fluorescence images from Ai9 mice treated with MC3-LNPs or P3B-LNPs encapsulating Cas9/sgTOM. n = 3 biological replicates. **(c)** Schematic highlighting distinct brain regions analyzed. **(d-f)** tdTomato expression in neurons, astrocytes, and microglia within the **(d)** hippocampus, **(e)** cortex, and **(f)** thalamus. Representative images selected from n = 3 mice per treatment group (biological replicates). Scale bar: 100 μm. **(g-i)** Quantification of tdTomato-positive neurons and astrocytes across the three brain regions. Two-tailed unpaired Student’s t-tests were used to determine significance. Data are presented as mean ± s.d.,n = 3 biological replicates.

Following the optimization of P3B-LNPs, we next assessed their ability to deliver functional gene-editing cargo to the brain. In line with the objectives of this study, we evaluated P3B LNPs using a CRISPR-Cas9 system rather than the Cre recombinase reporters commonly used in CNS delivery studies. While Cre reporters are valuable for initially assessing mRNA delivery, Cas9/sgRNA is a larger and more difficult payload that allows direct measurement of functional genome editing, which is central to establishing the therapeutic potential of P3B LNPs. To this end, we co-encapsulated Cas9 mRNA together with sgTOM, a guide RNA developed to target the LoxP-flanked stop cassette in Ai9 reporter mice (Fig. 3a). At baseline this cassette supresses tdTomato, but successful editing removes the stop sequence and activates tdTomato expression, providing a direct readout of Cas9-mediated genome editing *in vivo*. Ai9 mice received two IT injections of P3B or MC3 LNPs formulated CRISPR-Cas9 (Cas9 mRNA/sgTOM) (0.05 mg/kg mRNA/ mouse) one week apart, and brains were harvested on day 14 for analysis (Fig. 3a). Using this system, we aimed to assess the Cas9 delivery efficiency of P3B-LNPs to neurons and astrocytes across distinct brain regions (Fig. 3c). Compared with MC3 LNPs, P3B LNPs achieved robust and widespread tdTomato expression throughout the brain (Fig. 3b). Notably, P3B LNPs delivery extended beyond superficial layers into deep parenchymal tissue, demonstrating uniform access to cell populations that are typically inaccessible with systemic administration routes [23,26,27]. TdTomato expression was evident across the hippocampus, cortex, and thalamus, with P3B consistently outperforming MC3 in both neuronal and astrocytic populations (Fig. 3d–f). Quantitative analysis confirmed that P3B-LNPs achieved markedly higher tdTomato expression than MC3-LNPs across all examined brain regions (Fig. 3g–i, S18–21). In the hippocampus, P3B-LNPs transfected approximately 19.56 % of neurons, 20.21 % of astrocytes, and 17.74 % of microglia, representing roughly 2–3-fold increases over MC3-LNPs. In the cortex, tdTomato-positive cells reached 16.93 % in neurons, 19.52 % in astrocytes, and 12.56 % in microglia; and in the thalamus, P3B-LNPs achieved 12.46 % neuronal, 12.65 % astrocytic, and 14.36 % microglial transfection. All differences compared with MC3-LNPs were statistically significant (P < 0.01). For the spinal cord, P3B-LNPs yielded 19.14 % tdTomato^+^ neurons, ~22.75 % tdTomato^+^ astrocytes, and ~ 17.25 % tdTomato^+^ microglia, which were significantly higher than the MC3-LNP group (~7.45 %, ~6.85 %, and ~ 5.61 %, respectively; P = 0.0038, 0.0004, 0.0029; Fig. S20). The therapeutic importance of broad coverage is underscored by the regional specificity of CNS diseases. For example, Alzheimer’s disease is marked by hippocampal and cortical degeneration [48,49], whereas Parkinson’s disease involves dysfunctional thalamic circuits affecting motor control [50–52]. By achieving robust editing of both neurons and astrocytes across these regions, P3B-LNPs may provide wide therapeutic reach across diverse CNS pathologies.

### P3B LNPs enable ABE-mediated editing in the brain

Having established that P3B LNPs efficiently deliver Cas9 mRNA/sgRNA to the brain, we next evaluated their ability to support adenine base editor (ABE)-mediated gene editing. Unlike CRISPR-Cas9 complexes, which rely on double-strand breaks, ABEs enable precise single-nucleotide conversion, a more clinically relevant approach for correcting point mutations that underlie many neurological diseases [53]. To date, *in vivo* studies of base editing in the brain remain limited, with one report demonstrating perinatal brain ABE delivery via intracerebroventricular (ICV) injection for congenital disorders [54]. However, efficient ABE-mediated editing in the brain following IT administration, a clinically relevant and less invasive route, has not been demonstrated.

**Fig. 4.**
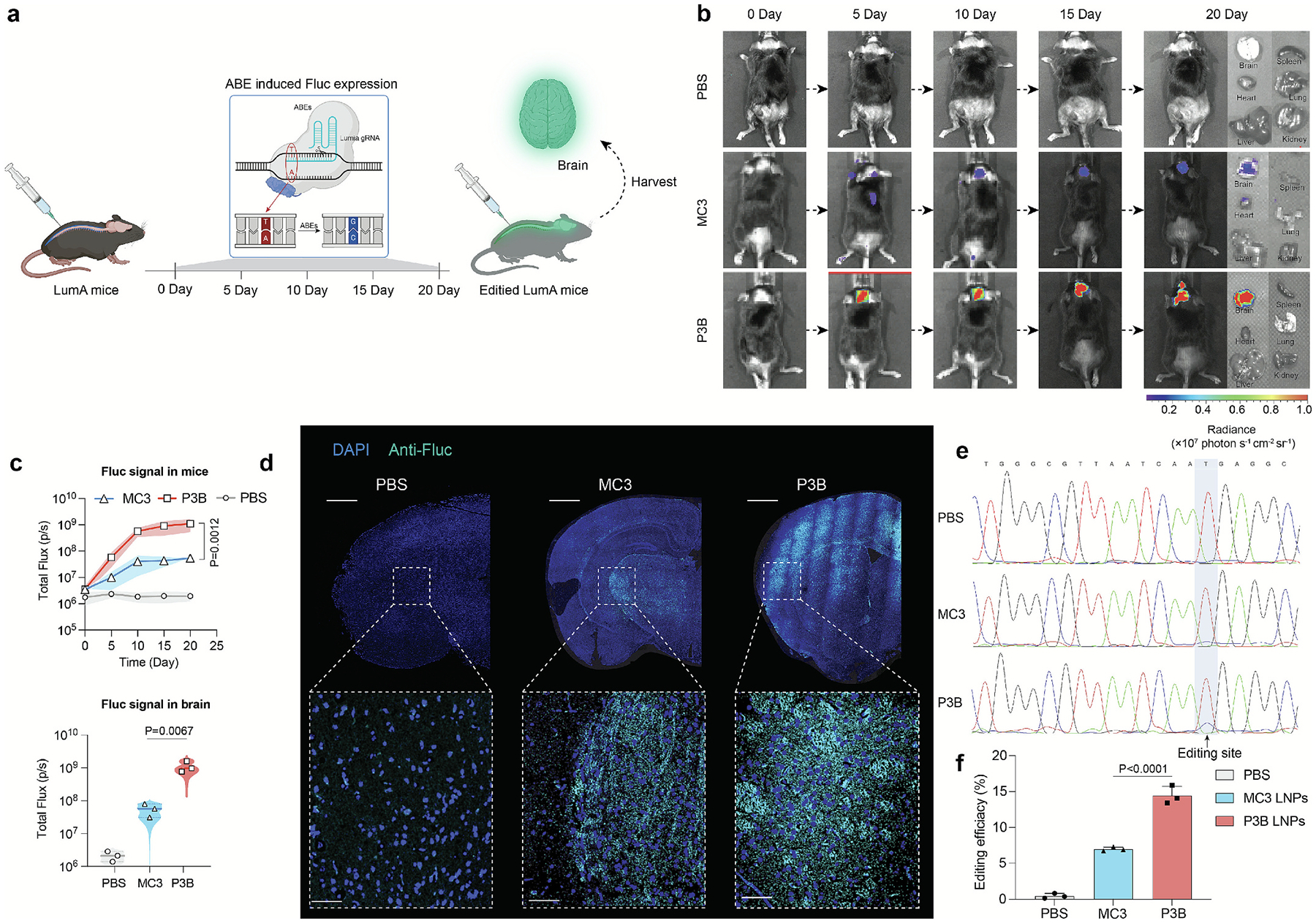
Intrathecal (IT) delivery of P3B encapsulated ABE enables brain base editing. **(a)** Schematic of ABE-mediated Fluc activation in LumA mice after intrathecal delivery. **(b)** Representative *in vivo* bioluminescence images of mice treated with PBS, MC3-LNPs or P3B-LNPs (0.05 mg/kg mRNA/mouse) over 20 days. n = 3 biological replicates. **(c)** Quantification of total Fluc signal in mice and brain tissue. Data represent mean ± s.d.; n = 3 mice per group (biological replicates). Two-tailed unpaired Student’s t-tests were used. **(d)** Immunofluorescence staining of brain sections showing DAPI (blue) and anti-Fluc (cyan) signals in LumA mice treated with PBS, MC3-LNPs or P3B-LNPs at day 20. Representative images selected from n = 3 mice (biological replicates). Scale bars, 1 mm (left) and 100 μm (right). **(e)** Sanger sequencing results confirming ABE-mediated base editing at the target site. **(f)** Quantification of Sanger sequencing results by EditR. n = 3 biological replicates. Data represent mean ± s.d.; one-way ANOVA multiple comparison test was used.

To evaluate this, we employed LumA (Luminescence ABE) reporter mice, which harbor a R387X point mutation in the luciferase gene at the ROSA26 locus that eliminates luciferase activity [55]. Successful ABE-mediated adenine-to-guanine (A-to-G) correction at the target site restores the coding sequence and induces Fluc expression, providing a direct readout of base editing *in vivo*. Following IT administration of MC3 or P3B LNPs encapsulated with ABE mRNA/sgRNA (0.05 mg/kg mRNA/mouse), editing outcomes were monitored at in 5 day intervals for 20 days (Fig. 4a). P3B-LNP-treated mice showed robust Fluc expression by day 5 that persisted through day 20, in contrast to the minimal activity observed with MC3 (Fig. 4b–c). Whole-animal quantification confirmed consistently elevated Fluc levels throughout the 20-day period, indicating durable genomic correction rather than transient Fluc expression. In the brain, P3B achieved an approximately 30-fold higher signal compared to the MC3 benchmark (Fig. 4c). *Ex vivo* analysis further supported this efficiency, with Fluc expression largely confined to the brain, suggesting a low incidence of off-target delivery. Consistent with these results, tissue-level analysis revealed widespread Fluc protein expression throughout the brain parenchyma in P3B-treated mice, surpassing MC3. To directly confirm editing at the DNA level, sequencing of the *Fluc* locus verified A-to-G conversion at the intended target site in P3B-treated mice (Fig. 4e). Quantification showed that P3B-LNPs (14.8 %) achieved significantly higher editing efficiency than MC3-LNPs (6.9 %) (Fig. 4f), confirming that Fluc activation resulted from precise nucleotide correction. Mechanistic imaging studies demonstrated that P3B-LNPs exhibited substantially reduced lysosomal colocalization relative to MC3-LNPs, indicating more efficient endosomal escape and cytosolic delivery of mRNA. This enhanced endosomal escape, together with the previously observed penetration across the CSF-brain interface, likely contributes to the superior CNS transfection efficiency of P3B-LNPs (Fig. S22–S23).

Together, these results demonstrate that P3B LNPs achieve efficient and specific ABE-mediated editing in the brain following IT administration. This capability is significant because base-editing offers a precise means of correcting pathogenic single-nucleotide variants without introducing double-strand breaks. For example, Rett syndrome is a neurodevelopmental disorder in which more than 60 % of cases arise from single point mutations in the *MECP2* gene, making it an ideal candidate for brain-targeted base editing [56,57]. By combining high efficiency with brain-restricted activity, IT-administered P3B LNPs represent a promising platform for advancing therapeutic base editing.

### Safety and tolerability of P3B LNPs

**Fig. 5.**
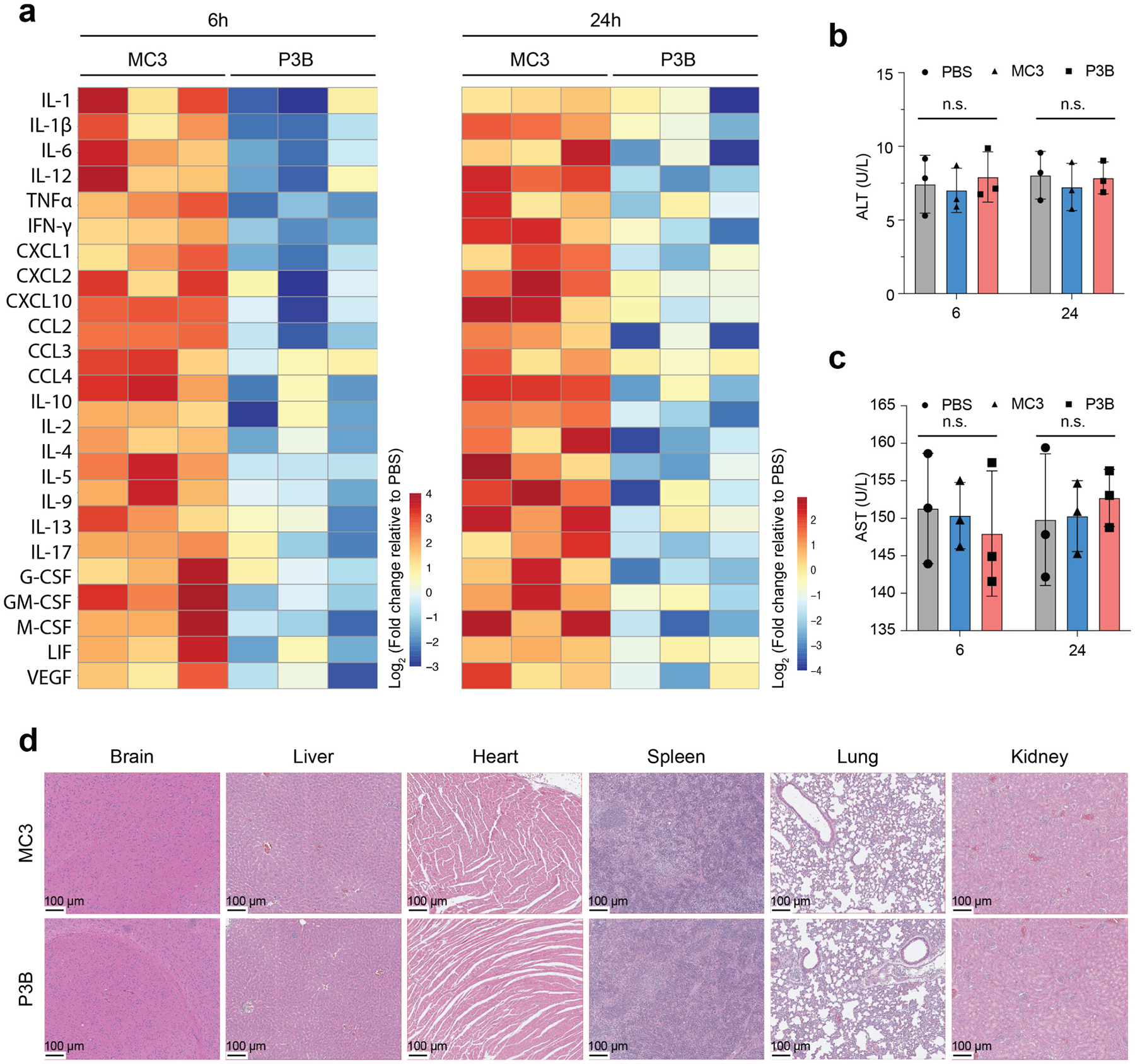
Safety evaluation of P3B LNPs *in vivo* after intrathecal (IT) dosing. (a) Heatmaps showing cytokine and chemokine expression in brains of mice 6 or 24 h after IT injection of P3B or MC3 LNPs (0.05 mg/kg mRNA/mouse), relative to PBS controls. (b-c) Plasma levels of alanine transaminase (ALT) and aspartate transaminase (AST) 6 or 24 h after IT injection. (d) Representative histopathological images of major organs (brain, liver, heart, spleen, lung, kidney) collected 6 h after IT injection (0.05 mg/kg mRNA/mouse), of MC3 or P3B LNPs. Scale bars, 100 μm. All data are from n = 3 biologically replicates and are presented as mean ± s. d. Statistical significance and P values were determined by one-way ANOVA multiple comparison test. n.s., not significant, P > 0.05.

Tolerability of brain-targeted materials is critical, as CNS parenchyma is highly vulnerable to injury and degeneration driven by prolonged inflammatory states [39,40]. To evaluate the safety of P3B LNPs following IT administration, plasma was collected at 6 h and 24 h post-injection for inflammatory biomarker profiling. Compared with MC3 LNPs, which triggered broad upregulation of cytokines and chemokines, P3B elicited a weaker response, with lower levels of key markers including interleukin-1 beta (IL-1β), interleukin 6 (IL-6), tumor necrosis factor alpha (TNF-α), interferon gamma (IFN-γ), and chemokines such as CXCL1, CCL2, and CCL3 (Fig. 5a). Notably, many markers were suppressed below baseline in P3B-treated mice, in contrast to the strong activation observed with MC3. By 24 h post injection, biomarker levels in the P3B group had largely returned to PBS control values, indicating a high level of tolerability (Fig. 5a). To assess hepatic function, plasma alanine aminotransferase (ALT) and aspartate aminotransferase (AST) were measured at 6 and 24 h post-injection. No significant changes were observed between groups (Fig. 5b–c), indicating that P3B LNPs do not cause detectable liver toxicity. Histopathological examination of major organs, including brain, liver, heart, spleen, lung, and kidney, revealed no evidence of tissue damage or inflammation at 6 h post-injection (Fig. 5d). Together, these findings demonstrate that P3B LNPs are well tolerated and exhibit a favourable safety profile for mRNA delivery to the brain via IT administration. This biocompatibility is consistent with the design principles of Passerini-derived lipids, which feature α-acyloxy amide scaffolds with hydrolyzable ester bonds [37]. These bonds promote biodegradation and clearance, thereby minimizing the potential adverse effects associated with lipid accumulation [45,58,59]. Metabolic stability assays using mouse liver microsomes further confirmed that P3B undergoes enzymatic cleavage of the Passerini-derived ester linkage, yielding three identifiable metabolites consistent with its designed biodegradability (Fig. S24). Together, these findings demonstrate that P3B LNPs are well tolerated and exhibit a favorable safety profile for intrathecal mRNA delivery to the brain. While these results establish excellent acute tolerability, the main limitation lies in the 24-hour evaluation window. Although these data confirmed minimal immune activation, comprehensive long-term studies involving repeated dosing and longitudinal monitoring are underway to further assess chronic safety, including potential neuroinflammatory and histopathological changes.

## Conclusion

In this study, we leveraged a high-throughput lipid synthesis and screening approach based on the Passerini reaction to identify ionizable lipids optimized for mRNA-based gene editor delivery to the CNS via intrathecal administration. From a 200-member library, we discovered P3B, a biodegradable ionizable lipid that enables efficient and widespread delivery of gene editors throughout the brain. Optimized P3B-LNP formulations achieved robust Cas9-mediated genome editing in both neurons and astrocytes across multiple brain regions, including the hippocampus, cortex, and thalamus, highlighting their broad potential for the treatment of CNS disorders. Moreover, P3B-LNPs supported precise adenine base editing, demonstrating their capacity for the targeted correction of single-nucleotide mutations underlying many neurological diseases. Across all applications, P3B outperformed the clinical benchmark MC3. Importantly, P3B-LNPs were biodegradable and well tolerated, with minimal systemic exposure and attenuated inflammatory responses, supporting their potential for safe, repeated dosing. This work highlights the power of combinatorial lipid design in advancing CNS-targeted genome editing and provides a versatile platform for refining lipid chemistry to address the unique delivery challenges of large, complex mRNA-based gene editors. Future efforts will priotize evaluating therapeutic efficacy and long-term safety of gene editing in CNS disease-relevant models.

## Materials and methods

### Lipid library and ionizable lipid synthesis

Ionizable lipids were synthesized using the Passerini three-component reaction (P-3CR), which couples carboxylic acids, aldehydes, and isocyanides in equimolar ratios (1:1:1) in dichloromethane [37]. Reactions were carried out in sealed 96-well PCR plates under ambient conditions for 24 h. Solvents were removed by vacuum drying, and crude lipids were resuspended in ethanol for high-throughput screening. The crude lipids were purified by flash chromatography (BUCHI). Structures of the synthesized lipids were confirmed by proton NMR (1H NMR) spectroscopy at 400 MHz (Bruker spectrometer).

### LNP formulation

LNPs were prepared by mixing an ethanolic lipid phase with an aqueous mRNA solution in a T-junction microfluidic device. After mixing, particles were dialyzed against PBS (10,000 MWCO cassette, Thermo Fisher) for 12 h at 4 °C. The lipid phase contained ionizable lipid, DSPC (Avanti), cholesterol (Avanti), and C14-PEG2000 (Avanti) at 50:10:38.5:1.5 M ratios, mirroring MC3 formulations. MC3 was obtained from Echelon Bioscience. The aqueous phase consisted of mRNA dissolved in 10 mM citrate buffer (pH 4.0). Final LNP suspensions contained 0.1 μg/μL mRNA.

Formulation optimization was performed to evaluate five key variables affecting LNP transfection efficiency and encapsulation: (1) ionizable lipid-to-mRNA mass ratio, (2) ionizable lipid molar fraction, (3) phospholipid type, (4) phospholipid molar fraction, and (5) PEG-lipid content. Experimental designs were generated using JMP13 software (SAS Institute).

### LNP characterization

The hydrodynamic diameter and polydispersity index (PDI) of LNPs were determined by dynamic light scattering (DLS) on a Malvern Nano ZS Zetasizer. LNP suspensions were diluted in phosphate-buffered saline (PBS, pH 7.4) to the appropriate mRNA concentration and loaded into disposable cuvettes. Measurements were carried out at 25 °C with a run time of 2 s per measurement, and the instrument was set to automatically adjust the number of runs and attenuation. Particle size distributions were reported as Z-average diameters.

mRNA concentration and encapsulation efficiency were quantified using the Quant-iT RiboGreen RNA assay kit (Invitrogen). To determine encapsulation efficiency, two measurements were performed. First, fluorescence was measured using intact LNPs, which reflects the amount of unencapsulated (free) RNA since RiboGreen cannot penetrate lipid membranes. Second, the same samples were treated with 1 % Triton X-100 to disrupt the LNPs and release encapsulated RNA, providing the total RNA signal. Encapsulation efficiency was calculated as (Total RNA − Free RNA) / Total RNA × 100 %.

### Animal experiments

All experimental procedures are ethically approved, and all animal studies were approved and conducted in compliance with the University Health Network Animal Resources Centre guidelines. Female mice (6 to 8 week) BALB/C, B6. Cg-Gt (ROSA)^26Sortm9(CAG-tdTomato) Hze^/J (Ai9), and C57BL/6J-Gt^(ROSA)26Sorem1Crx^/J (LumA) were obtained from the Jackson Laboratory.

### In vivo batch-based testing

For *in vivo* batch testing, LNP mixtures within each structural class were pooled, dialyzed, and administered intrathecally to mice. Then, a uniform dose of 0.05 mg/kg mFluc-LNPs was used across all stages: 25 mixtures per group in the first-round batch analysis, 5 mixtures per group in the second round, and single formulations in the third round. At 6 h after injection, mice were subjected to the bioluminescence assay using an *in vivo* imaging system (IVIS kinetic imaging system, Perkin Elmer).

### Bioluminescence analysis

BALB/c mice (6–8 weeks, 20 ± 2 g) were injected intrathecally with mFluc-LNPs (0.05 mg/kg). At 6 h post-administration, animals received an intraperitoneal injection of D-luciferin (0.2 mL, 10 mg/mL in DPBS; PerkinElmer). After 10 min, mice were anesthetized with 1.5 % isoflurane in oxygen and imaged using an IVIS system (PerkinElmer). Brains were then harvested for *ex vivo* imaging, and luminescence signals were quantified using Living Image software (PerkinElmer).

### Cryogenic Transmission Electron Microscopy of LNPs

Concentrated LNP suspensions (10–20 mg/mL lipid) were applied as small drops onto polymer-coated, carbon-reinforced copper grids, blotted with filter paper to form thin films, and vitrified in liquid ethane at −180 °C. Grids were prepared immediately before imaging and maintained below −165 °C. Samples were imaged on a FEI Tecnai F20 TEM (Thermo Fisher Scientific, 200 kV) equipped with a Gatan K2 Summit detector. Images were acquired at 25,000× magnification in counting mode (pixel size 1.45 Å; ~5 e^−^/pixel/s) with automated collection (EPU software). Image analysis was performed using iTEM (Olympus), and particle size distributions were calculated from ≥ 3 high-quality images per sample, with > 100 particles analyzed for each formulation.

### mRNA and single guide RNA (sgRNA)

ABE8e-SpRY mRNA was generated by linearizing ABE plasmids with NdeI (NEB, #R0111), purifying DNA fragments with the FastPure Gel Extraction Kit (Vazyme, #DC301), and performing *in vitro* transcription using the HiScribe T7 RNA Synthesis Kit (NEB, #E2040) with N1-methyl-pseudouridine-5′-triphosphate (SyngeneBio) substituted for UTP. Transcripts were capped using the Faustovirus capping enzyme (NEB, #M2081) and 2′-O-methyltransferase (NEB, #M0366), followed by polyadenylation with E. coli poly(A) polymerase (NEB, #M0276). Final products were purified, quantified by NanoDrop, and adjusted to 1 μg/μL for LNP encapsulation. Fully modified mRNAs encoding Fluc (TriLink, #L-7602), Cre (TriLink, #L-7603), and Cas9 (TriLink, #L-8106), with CleanCap m6AG 3′OMe chemistry, were purchased from TriLink. sgRNAs carrying 2′-O-methyl and phosphorothioate modifications at the first and last three nucleotides were obtained from IDT, with sequences provided in Supplementary Table 2.

### Gene editing in Ai9, LumA reporter mice

Ai9 mice received weekly intrathecal injections of LNPs encapsulating mCas9 and sgTOM (3:1 M ratio; total RNA dose, 0.05 mg/kg per mouse) for two consecutive weeks. Brains were collected one week after the final injection for downstream analyses.

LumA mice were administered intrathecal injections of LNPs containing mABE and sgRNA (1:1 ratio; 0.05 mg/kg per mouse) on day 0, with a total of three doses. Luciferase activity was monitored by IVIS imaging over 16 days, after which mice were sacrificed and brains and major organs were harvested for luminescence analysis.

Collected tissues were fixed in 4 % PFA and processed at the STTARR facility (UHN) for sectioning, staining, and imaging.

### Cell isolation and staining for flow cytometry

Harvested brains were digested in serum-free DMEM containing Collagenase IV (BioShop) for 1 h at 37 °C and passed through a 70 μm strainer to obtain single-cell suspensions. One million cells per sample were transferred to 1.5 mL tubes and stained with Zombie NIR viability dye (1:1000, BioLegend). Cells were then blocked with 1 % BSA/1% FBS and incubated with anti-NeuN and anti-GFAP antibodies (BioLegend) according to the manufacturer’s instructions. Finally, cells were fixed in 4 % PFA and analyzed by flow cytometry.

### Data analysis

Statistical analyses were conducted using GraphPad Prism 9. Two-tailed unpaired Student’s t-tests were used to determine significance. Data are presented as mean ± s.d., with significance levels denoted as: *P < 0.01, **P < 0.01, ***P < 0.001, and ****P < 0.0001.

## Supplementary Material

1

## Data Availability

Data will be made available on request.

## Appendix A. Supplementary data

Supplementary data to this article can be found online at https://doi.org/10.1016/j.mattod.2025.11.032.
