# Supplementary material for: Biodegradable lipid nanoparticles for genome editing in the brain via intrathecal administration
Source: Mater Today (Kidlington). Author manuscript; Available in PMC 2026 Jul 2. (PMC13322347; doi:10.1016/j.mattod.2025.11.032)
Supplement: 1 [file NIHMS2187312-supplement-1.docx]

**Biodegradable Lipid Nanoparticles for Genome Editing in the Brain via Intrathecal Administration**

Songtao Dong^1#^, Lauren Healy^2#^, Fanglin Gong^3^, Yue Xu^1^, Yunshu Cai^1,2^, Nicholas C. Solek^3^, Jingan Chen^3^, Muye Zhou^1^, Tyler Thomson^3^, Margarita Savguira^3^, Sijin Luozhong^1^, Yanchao Zhang^1^, Tingzhen He^3^, Gen Li^3^, Bowen Li^1,2,3*^

^1^ Leslie Dan Faculty of Pharmacy, University of Toronto, Toronto, Ontario, M5S 3M2, Canada.

^2^ Department of Chemistry, University of Toronto, Toronto, Ontario, M5G 1L7, Canada.

^3^ Institute of Biomedical Engineering, University of Toronto, Toronto, Ontario, M5S 3G9, Canada.

^#^ These authors contributed equally.

^*^ Communication can be sent to [bw.li@utoronto.ca](mailto:bw.li@utoronto.ca)

Table of Contents

[Section A. Supplementary Methods 3](#_Toc207361346)

[Section B. Characterization Data 3](#_Toc207361347)

[Section C. Supplementary Figures and Tables 11](#_Toc207361348)

[Section D. References 16](#_Toc207361349)

# Section A. Supplementary Methods

***Synthesis of combinatorial ionizable lipid library.***

Headgroups H1-H8 and Tails B1-B4 were purchased from commercial suppliers and used without further purification. Crude products were purified by flash column chromatography on a BUCHI CombiFlash system, affording materials suitable for NMR characterization. ^1^H NMR spectra were recorded on Bruker AVANCE II 400 MHz. All spectra were acquired in CDCl₃, and chemical shifts are reported in ppm relative the residual solvent peak (δ = 7.26 ppm).

*General Synthesis of Tail A*

Tail A structures were synthesized as previously described in the literature [1]. A primary amine (R–NH₂, 1.0 equiv), sodium chlorodifluoroacetate (2.0 equiv), and potassium carbonate (2.2 equiv) were combined in a minimal volume of dry N,N-dimethylformamide under a nitrogen atmosphere with a reflux condenser. The reaction mixture was stirred under nitrogen at 100 °C for 12 h. After cooling to room temperature, the mixture was extracted with dichloromethane, and the combined organic layers were washed with large volumes of water (×4) and brine (×1), then dried over anhydrous magnesium sulfate. The solvent was evaporated under a vaccum, and the crude residue was purified by flash column chromatography on a CombiFlash system (0–50% ethyl acetate in hexanes) to afford the desired isocyanide tails (TA1–TA5).

*Synthesis of Tail B5*

Synthesized as previously described [2]. cis,cis-9,12-octadecadien-1-ol was dissolved in dichloromethane under a nitrogen atmosphere, and Dess–Martin periodinane (2.0 equiv) was added. The mixture was stirred at room temperature for 2 h, and completion of the reaction was confirmed by thin-layer chromatography. An aqueous solution of sodium thiosulfate (50% w/v, 200 ml) was then added, and the mixture was stirred for an additional 15 min. The organic layer was separated, washed with brine, dried over anhydrous magnesium sulfate, and concentrated under reduced pressure. The crude residue was purified by automated flash column chromatography on a CombiFlash system (0–20% ethyl acetate in hexanes) to afford (9Z,12Z)-octadeca-9,12-dienal (TB5).

*Synthesis of P3B Lipid*

P3B was synthesized by a three-component Passerini reaction as previously described [2]. A mixture of 2-(1-methylpiperidin-4-yl)acetic acid (H8, 1.0 equiv), 1-isocyanotetradecane (TA2, 1.0 equiv), and (9Z,12Z)-octadeca-9,12-dienal (TB5, 1.0 equiv) was dissolved in anhydrous dichloromethane and stirred in capped glass vials at room temperature for 12 h. The crude mixture was concentrated under reduced pressure and purified by flash column chromatography on a CombiFlash system (0-10% MeOH in dichloromethane) to yield P3B as a colourless oil. The purity of P3B is >95% (Fig. S10-11).

#

# Section B. Characterization Data

*Tail A1 (1-isocyanohexadecane)*

^1^H NMR (400 MHz, CDCl_3_) δ 3.41 – 3.33 (m, 2H), 1.73 – 1.61 (m, 2H), 1.48 – 1.37 (m, 2H), 1.36 – 1.20 (m, 24H), 0.88 (t, 3H).


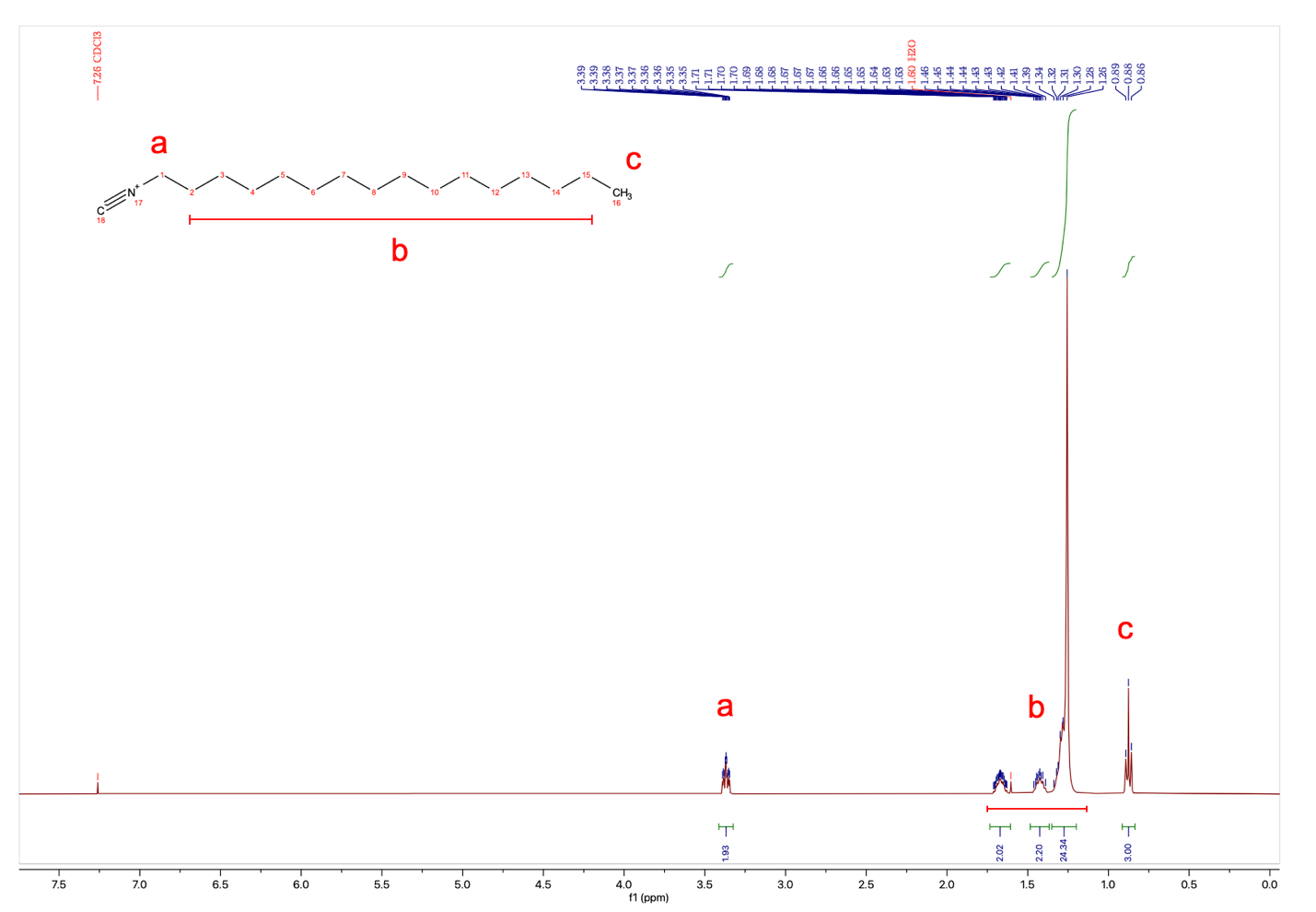


**Figure S1**. ^1^H NMR Spectrum for Tail A1

*Tail A2 (1-isocyanotetradecane)*

^1^H NMR (400 MHz, CDCl_3_) δ 3.41 – 3.33 (m, 2H), 1.74 – 1.61 (m, 2H), 1.48 – 1.37 (m, 2H), 1.34 – 1.22 (m, 20H), 0.87 (t, 3H).


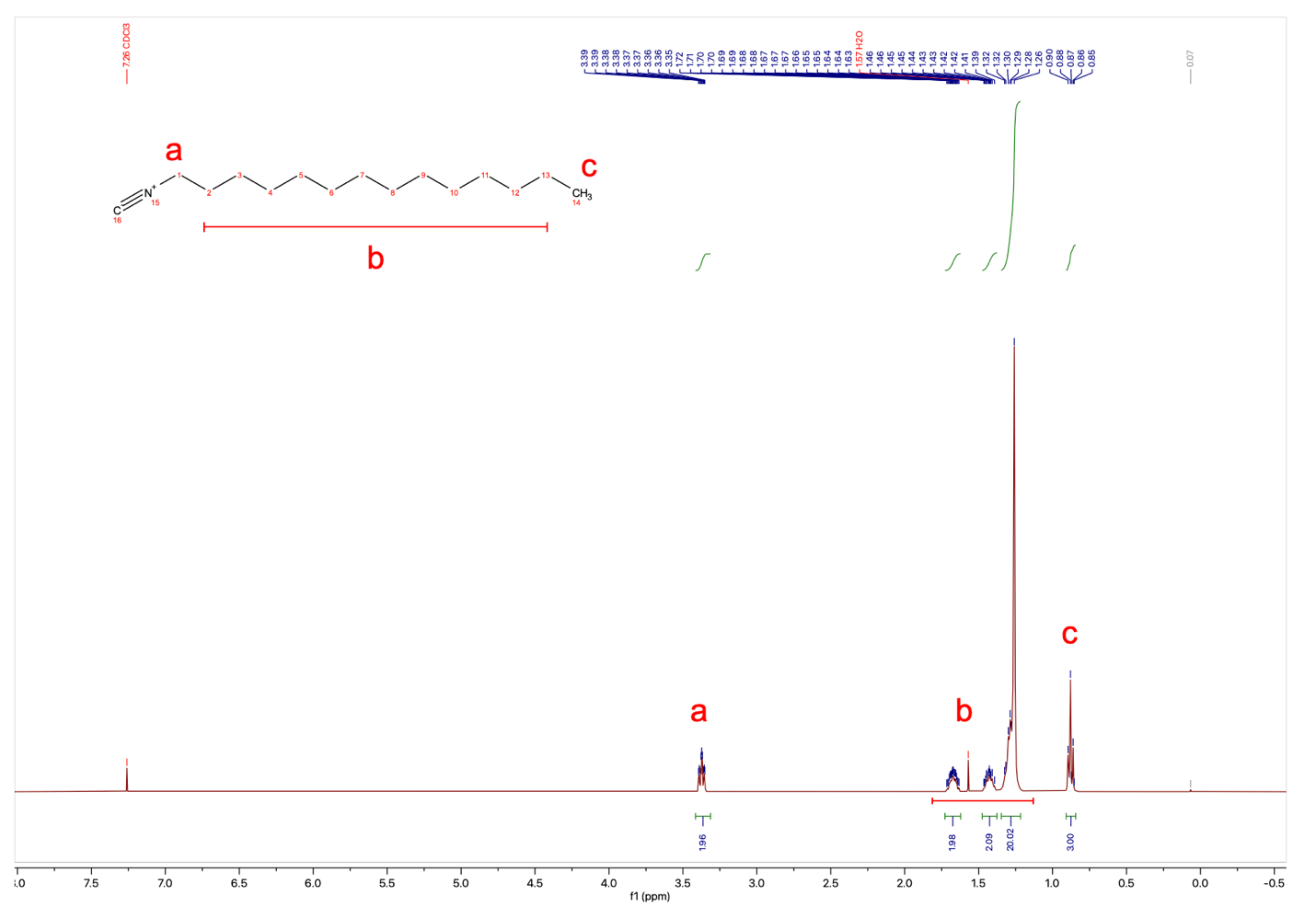


**Figure S2**. ^1^H NMR Spectrum for Tail A2

*Tail A3 (1-isocyanododecane)*

^1^H NMR (400 MHz, CDCl_3_) δ 3.41 – 3.33 (m, 2H), 1.73 – 1.59 (m, 2H), 1.48 – 1.37 (m, 2H), 1.36 – 1.24 (m, 16H), 0.88 (t, 3H).


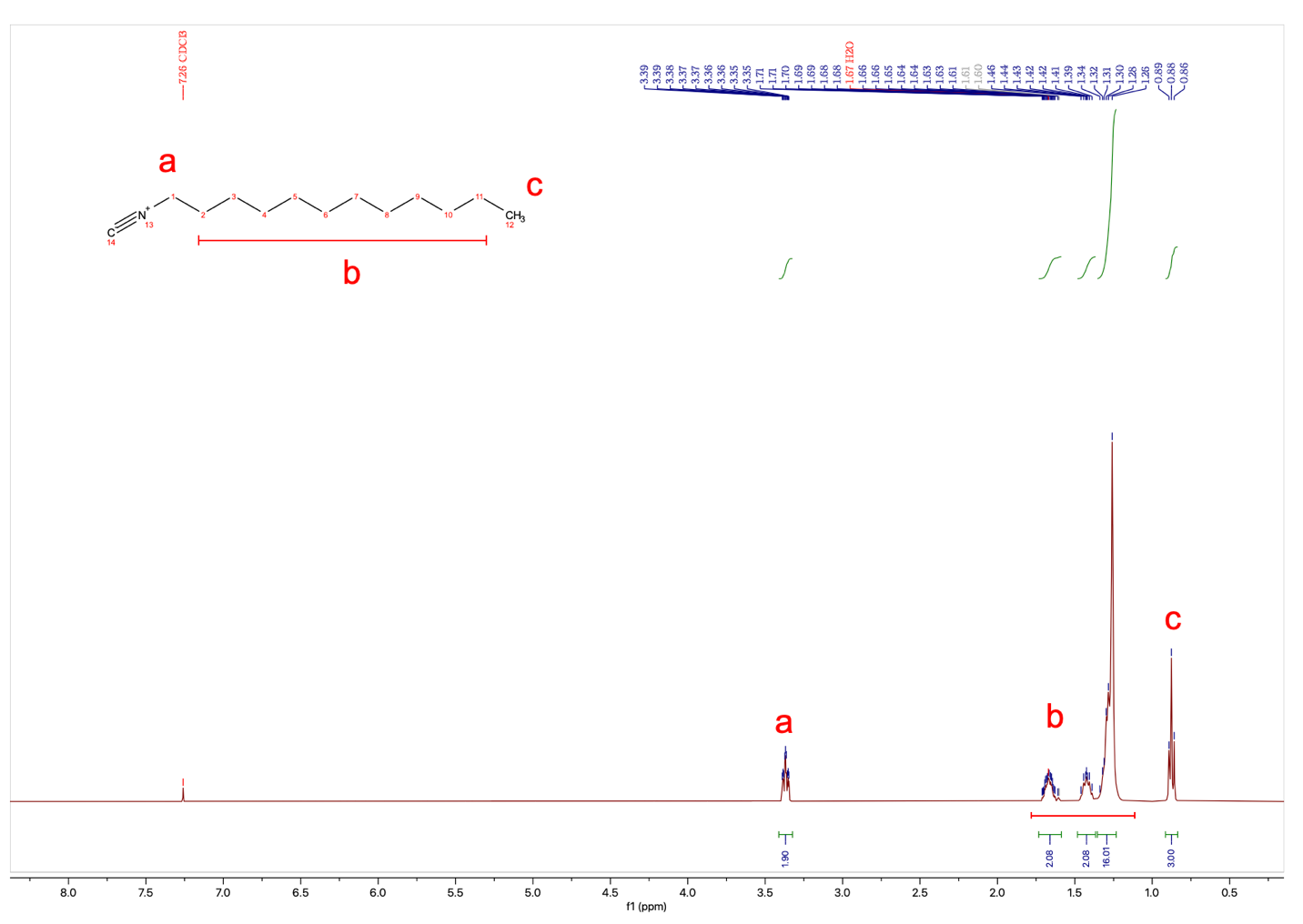


**Figure S3**. ^1^H NMR Spectrum for Tail A3

*Tail A4 (1-isocyanooctadecane)*

^1^H NMR (400 MHz, CDCl_3_) δ 3.41 – 3.33 (m, 2H), 1.74 – 1.61 (m, 2H), 1.43 (h, *J* = 4.6 Hz, 3H), 1.34 – 1.22 (m, 28H), 0.88 (t, 3H).


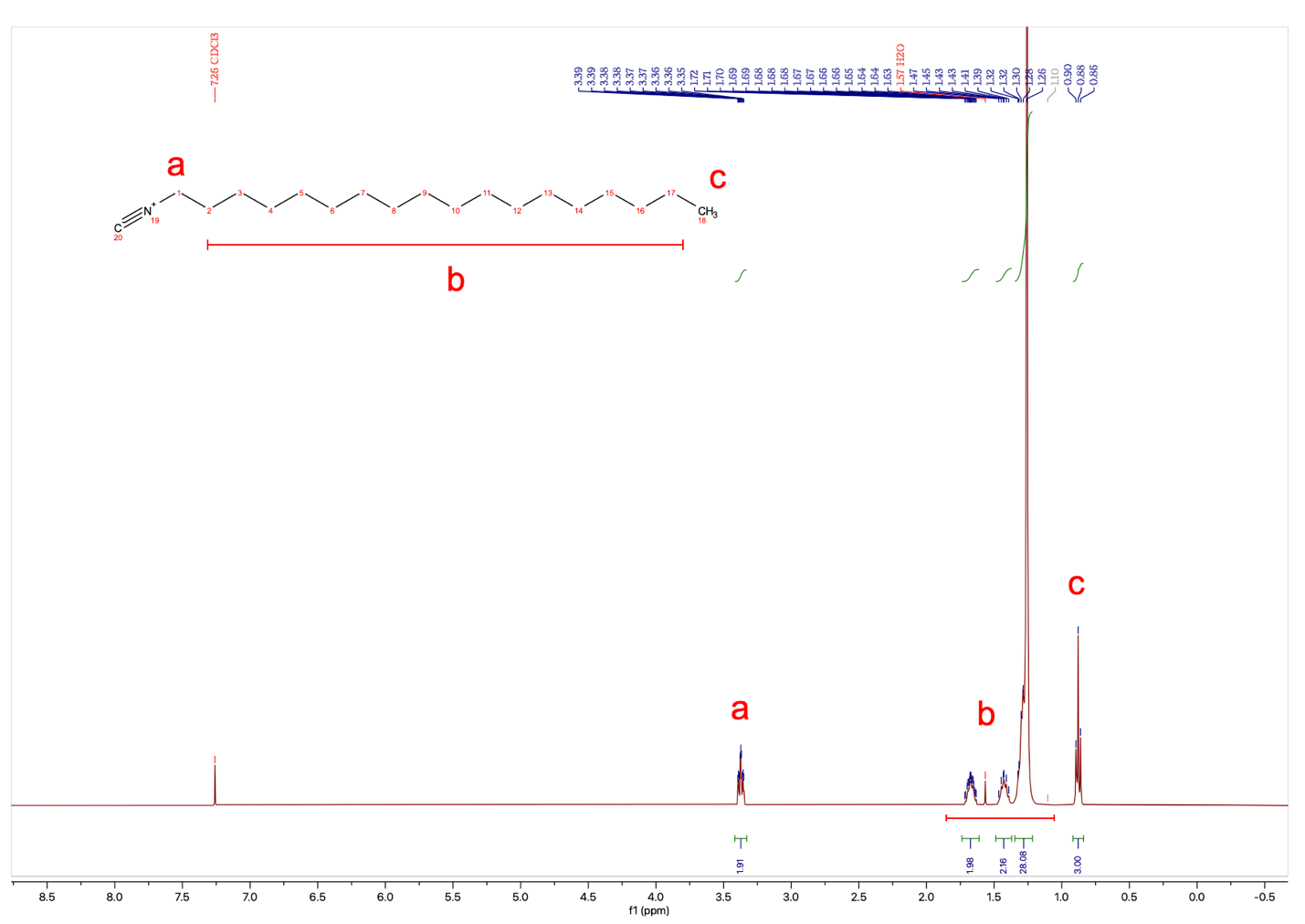


**Figure S4**. ^1^H NMR Spectrum for Tail A4

*Tail A5 ((Z)-1-isocyanooctadec-9-ene)*

^1^H NMR (400 MHz, CDCl_3_) δ 5.43 – 5.29 (m, 2H), 3.41 – 3.32 (m, 2H), 2.08 – 1.92 (m, 4H), 1.73 – 1.58 (m, 2H), 1.43 (h, *J* = 4.0 Hz, 2H), 1.34 – 1.23 (m, 20H), 0.87 (t, 3H).


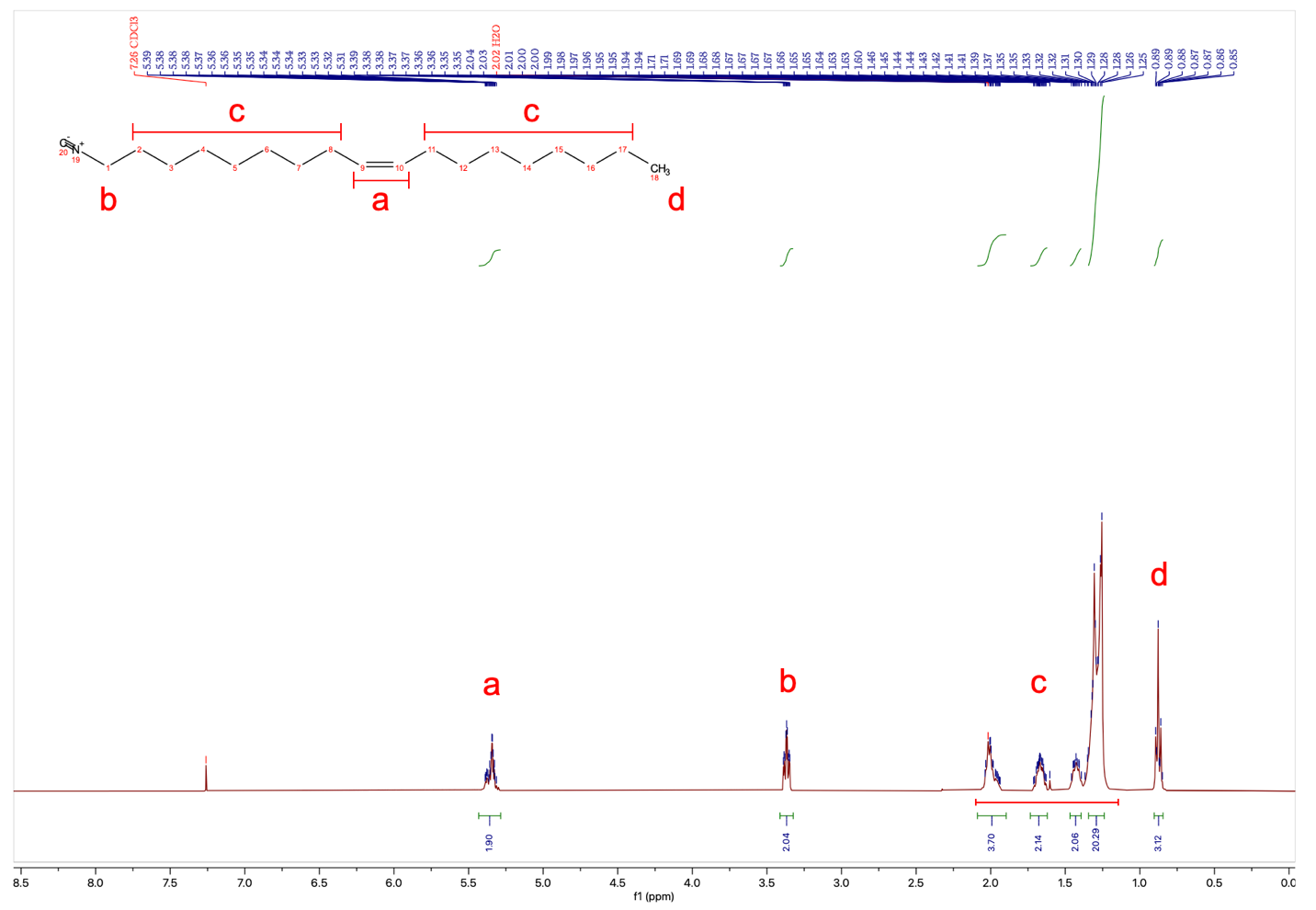


**Figure S5**. ^1^H NMR Spectrum for Tail A5

*Tail B5 ((9Z,12Z)-octadeca-9,12-dienal)*

^1^H NMR (400 MHz, CDCl_3_) δ 9.76 (t, *J* = 1.9 Hz, 1H), 5.44 – 5.27 (m, 4H), 2.77 (t, *J* = 5.7 Hz, 2H), 2.42 (td, *J* = 7.4, 1.9 Hz, 2H), 2.05 (q, *J* = 6.9 Hz, 4H), 1.62 (q, *J* = 7.2 Hz, 2H), 1.42 – 1.24 (m, 14H), 0.89 (t, 3H).


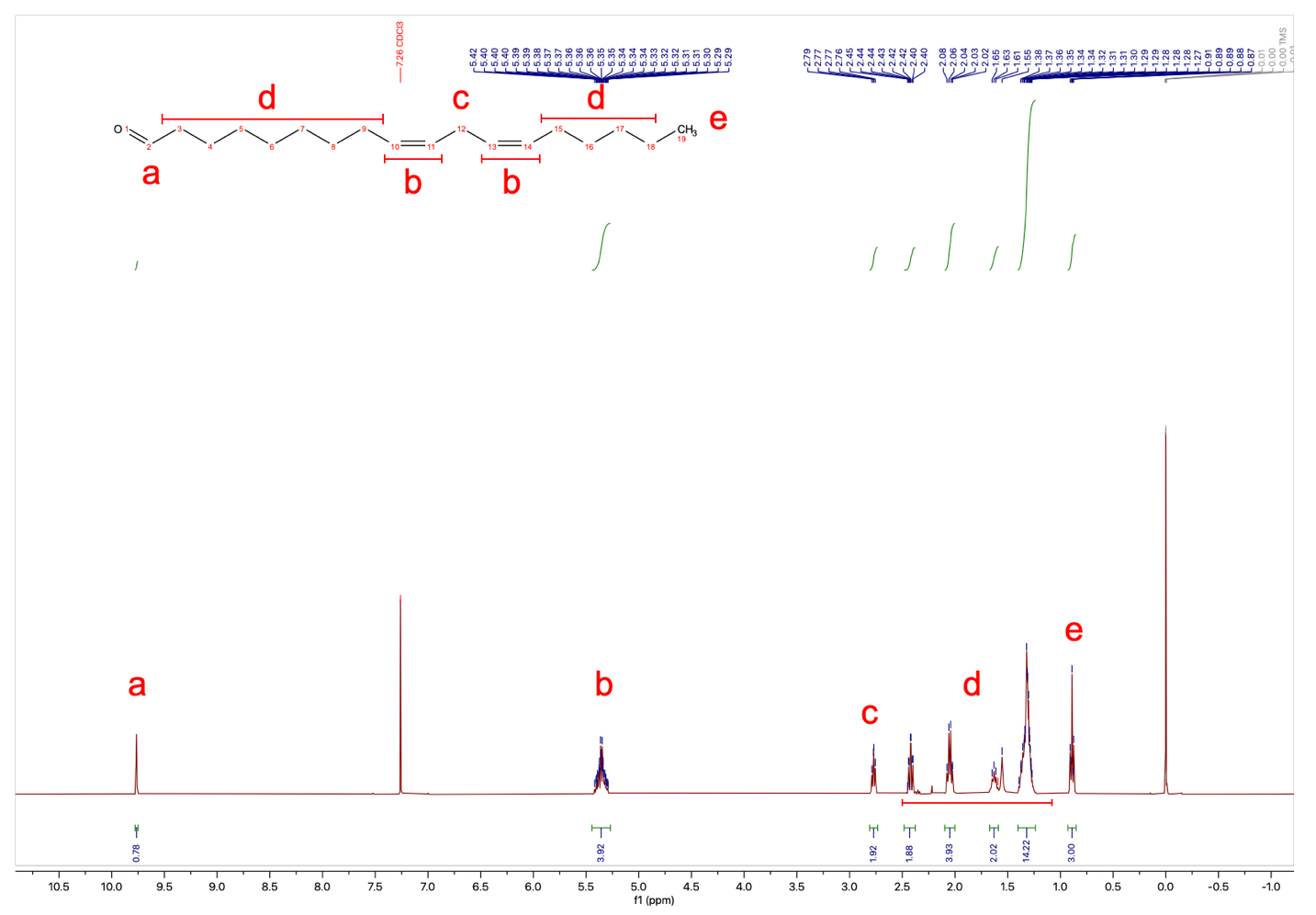


**Figure S6**. ^1^H NMR Spectrum for Tail B5

*P-3CR–Brain (P3B) ((10Z,13Z)-1-oxo-1-(tetradecylamino)nonadeca-10,13-dien-2-yl 2-(1-methylpiperidin-4-yl)acetate)*

^1^H NMR (400 MHz, CDCl_3_) δ 5.94 (t, *J* = 5.9 Hz, 1H), 5.43 – 5.31 (m, 4H), 5.17 – 5.12 (m, 1H), 3.25 (qd, *J* = 7.1, 2.2 Hz, 2H), 2.96 (d, *J* = 11.5 Hz, 2H), 2.77 (t, *J* = 6.5 Hz, 2H), 2.36 (s, 3H), 2.34 (s, 1H), 2.11 – 2.01 (m, 6H), 1.85 – 1.77 (m, 4H), 1.53 – 1.43 (m, 4H), 1.34 – 1.21 (m, 40H), 0.88 (td, *J* = 6.9, 4.0 Hz, 6H).


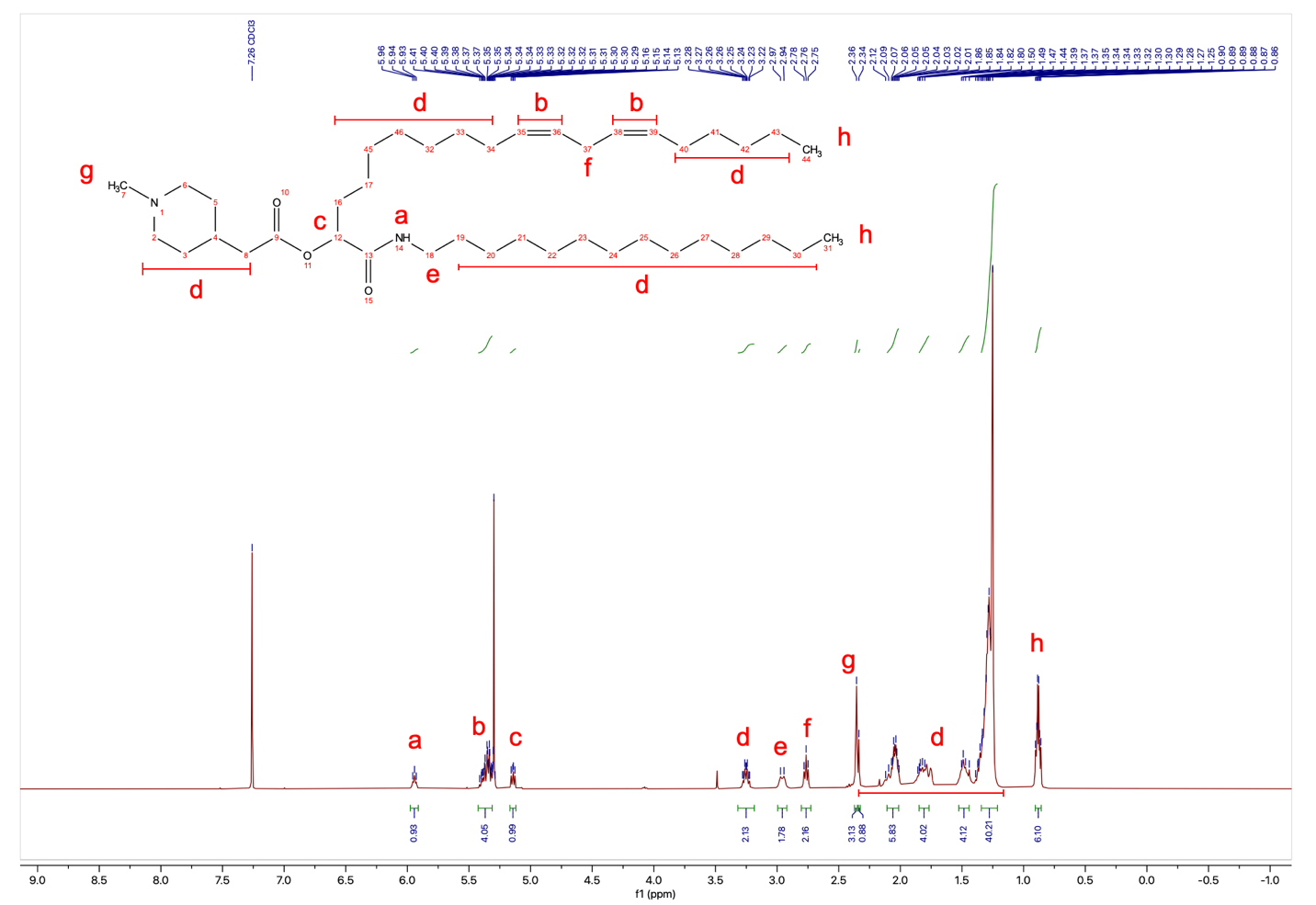


**Figure S7**. ^1^H NMR Spectrum for P-3CR–Brain (P3B)


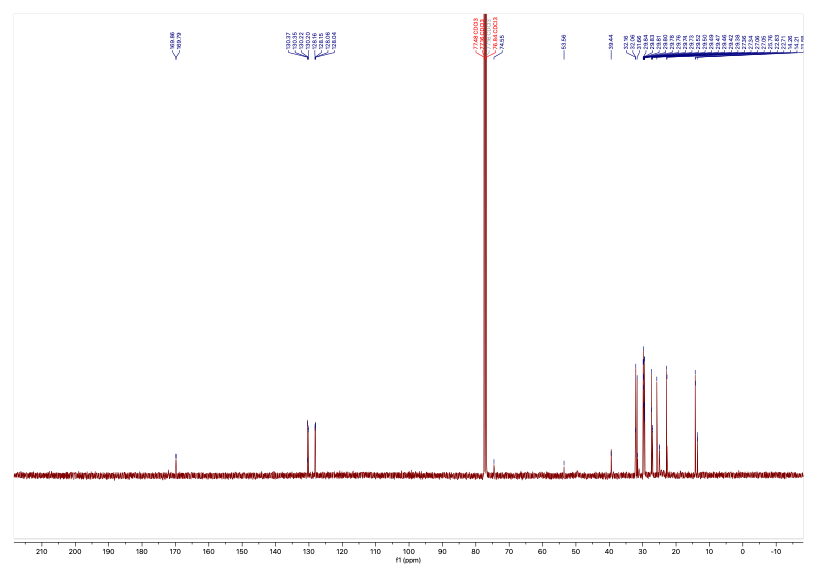


**Fig. S8.** The C NMR of P3B lipid.


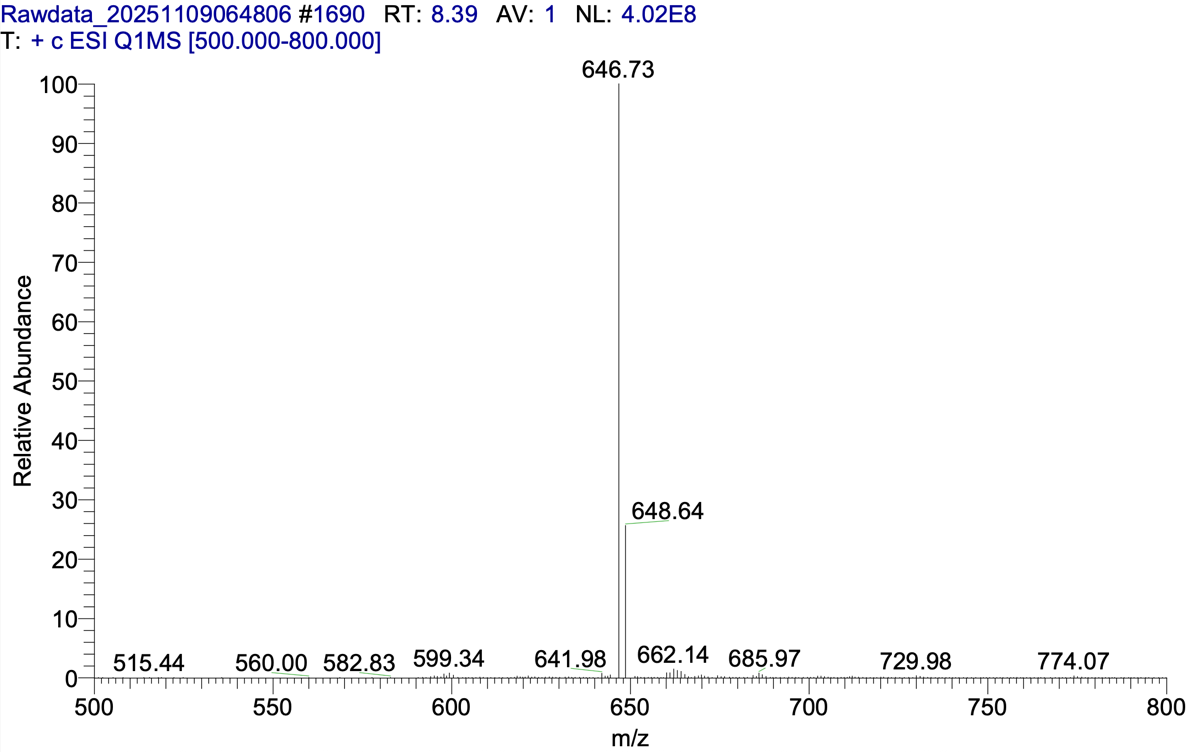


**Figure S9**. Mass Spectrum for P3B lipid.


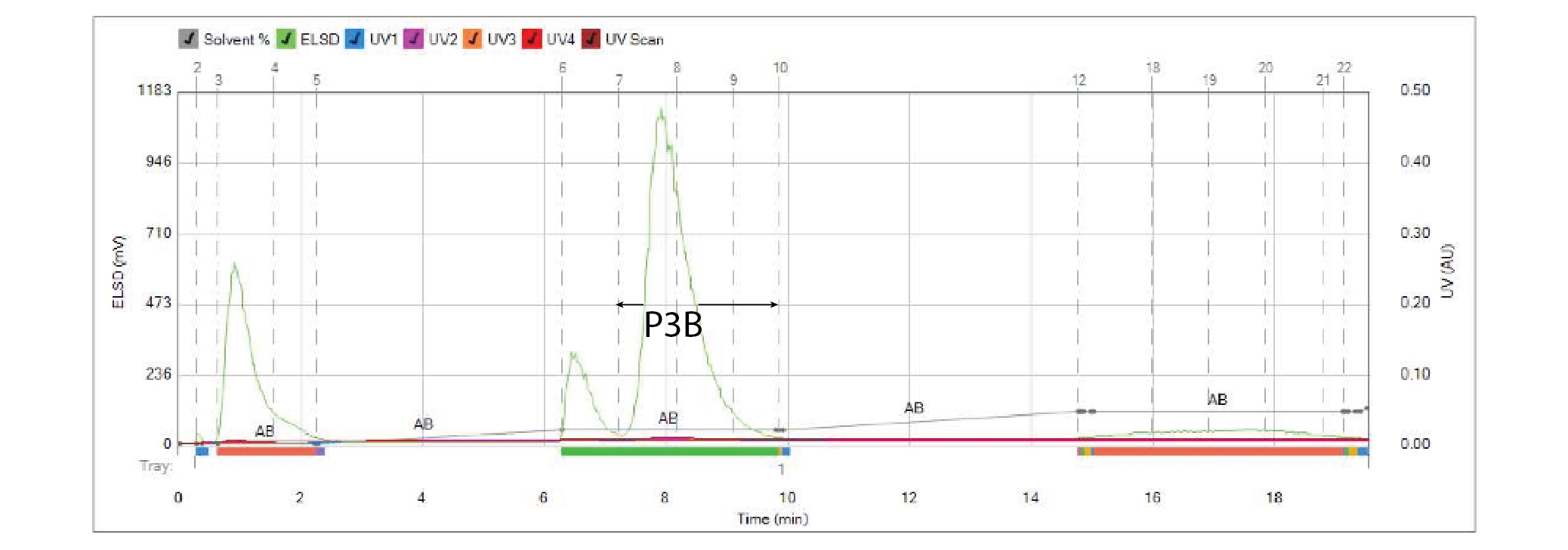


**Figure S10.** The purification of P3B lipid. MeOH/DCM=0-10% in 20 mins.


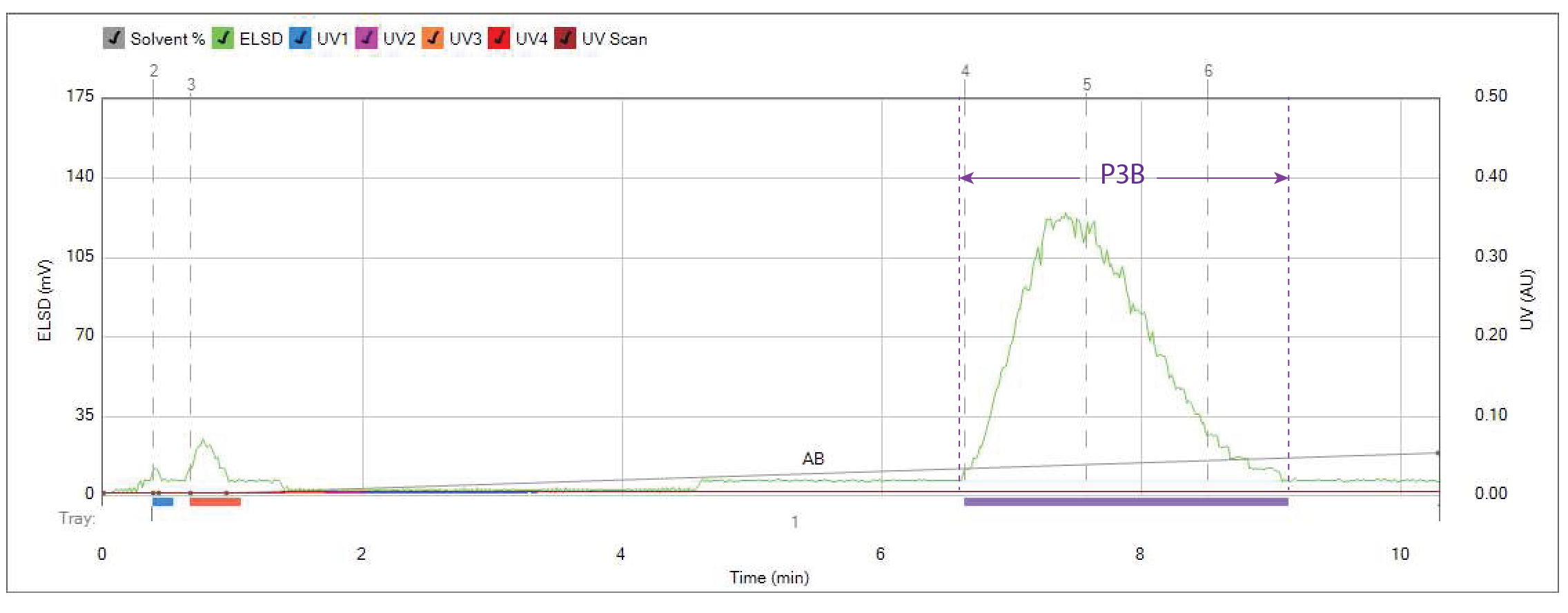


**Figure S11.** The purity of P3B lipid.

# Section C. Supplementary Figures and Tables

**Figure S12.** Quantification of Fluc luminescence in the brains of mice shown in the Figure 1c. (Round 1 screening of Tail A). Bar graphs display mean and standard deviation (S.D.) values from n=3 biological replicates. Statistical significance was determined by one-way ANOVA with multiple comparisons.

**Figure S13.** Quantification of Fluc luminescence in the brains of mice shown in the Figure 1d (Round 1 screening of Tail B). Bar graphs display mean and standard deviation (S.D.) values from n=3 biological replicates. Statistical significance was determined by one-way ANOVA with multiple comparisons.

**Figure S14.** Quantification of Fluc luminescence in the brains of mice shown in the Figure 1e (Round 3 screening of Headgroups). Bar graphs display mean and standard deviation (S.D.) values from n=3 biological replicates. Statistical significance was determined by one-way ANOVA with multiple comparisons.


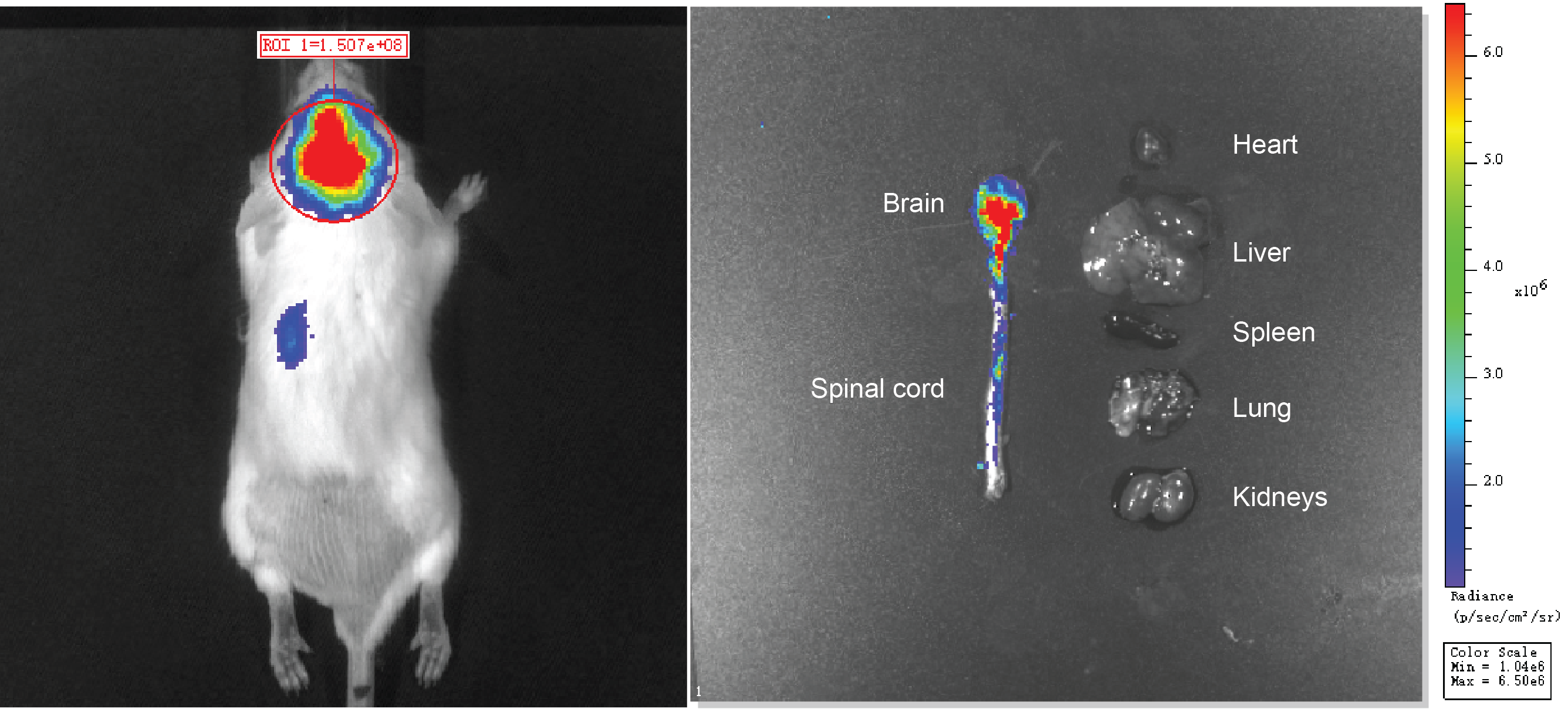


**Figure S15**. Representative *in vivo* and *ex vivo* bioluminescence imaging 6 hours after intrathecal (IT) administration of P3B-LNPs encapsulating firefly luciferase mRNA.

**Figure S16.** Determination of the apparent pKa of P3B LNPs by TNS fluorescence assay.


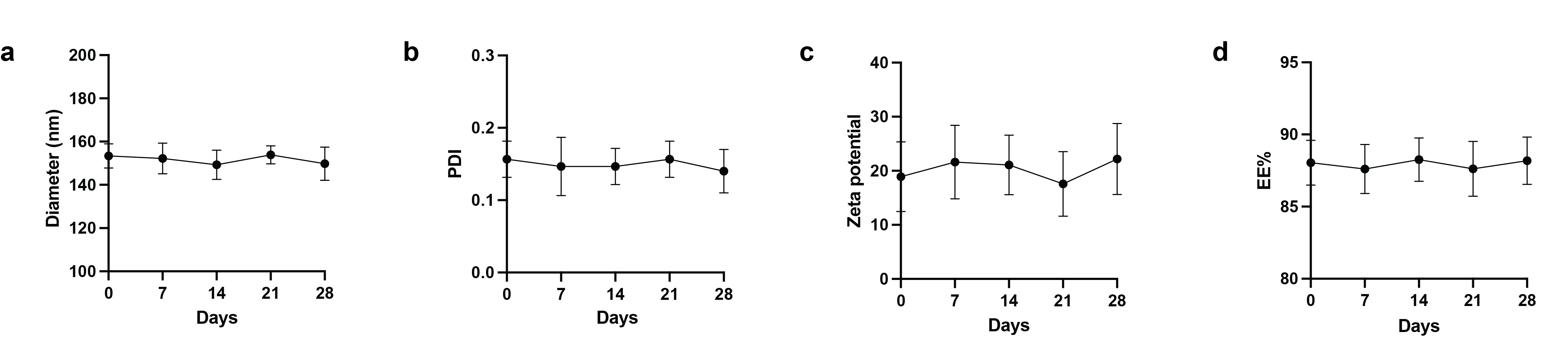


**Figure S17.** Stability of LNP formulations during long-term storage in PBS at 4 °C. **(a)** Average particle diameter, **(b)** polydispersity index (PDI), **(c)** zeta potential, and **(d)** encapsulation efficiency (EE%) of LNPs were monitored over 28 days. Data are presented as mean ± s.d. (n = 3).


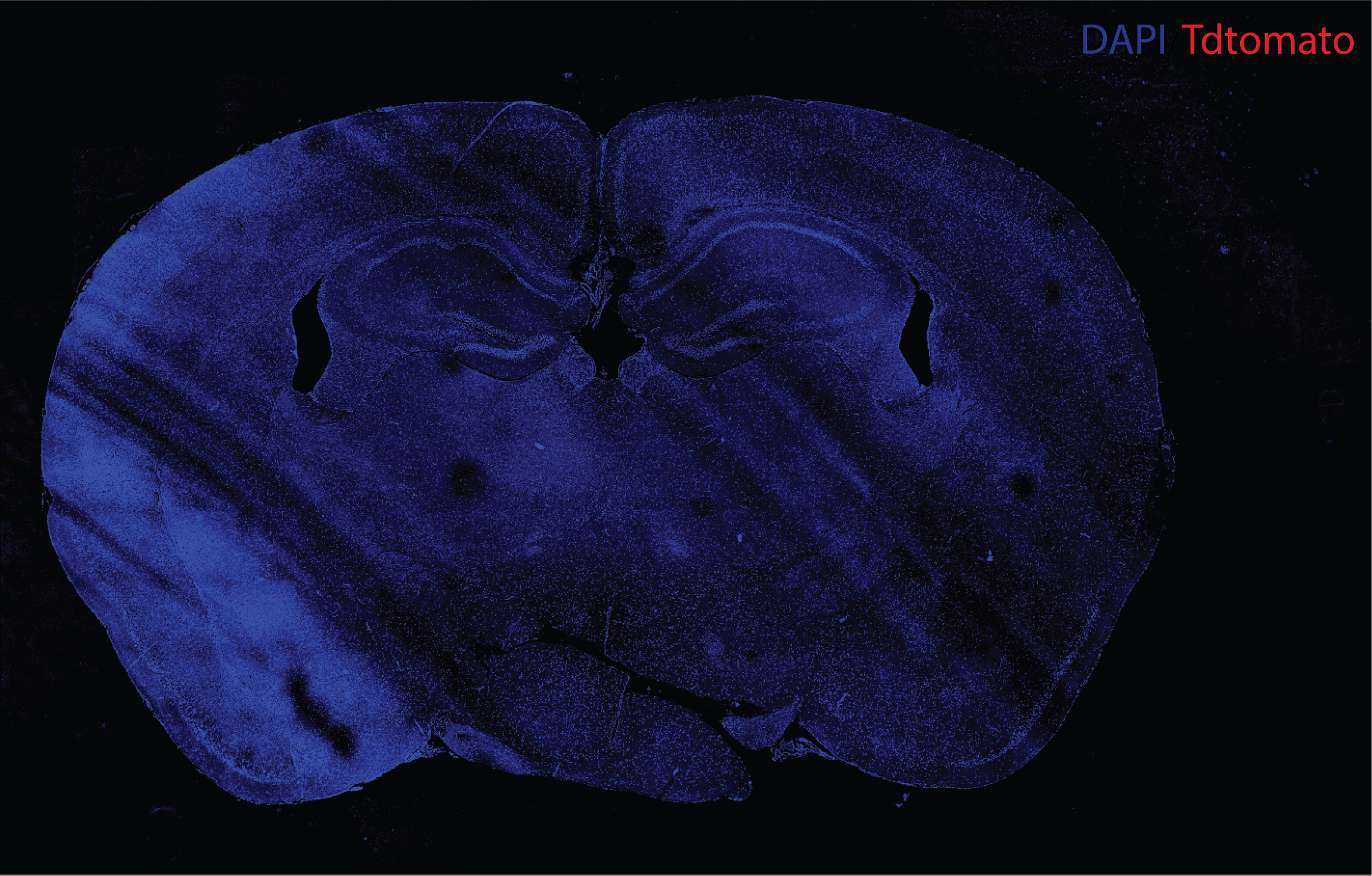


**Figure S18**. Representative brain image of Ai9 mice in the PBS group.


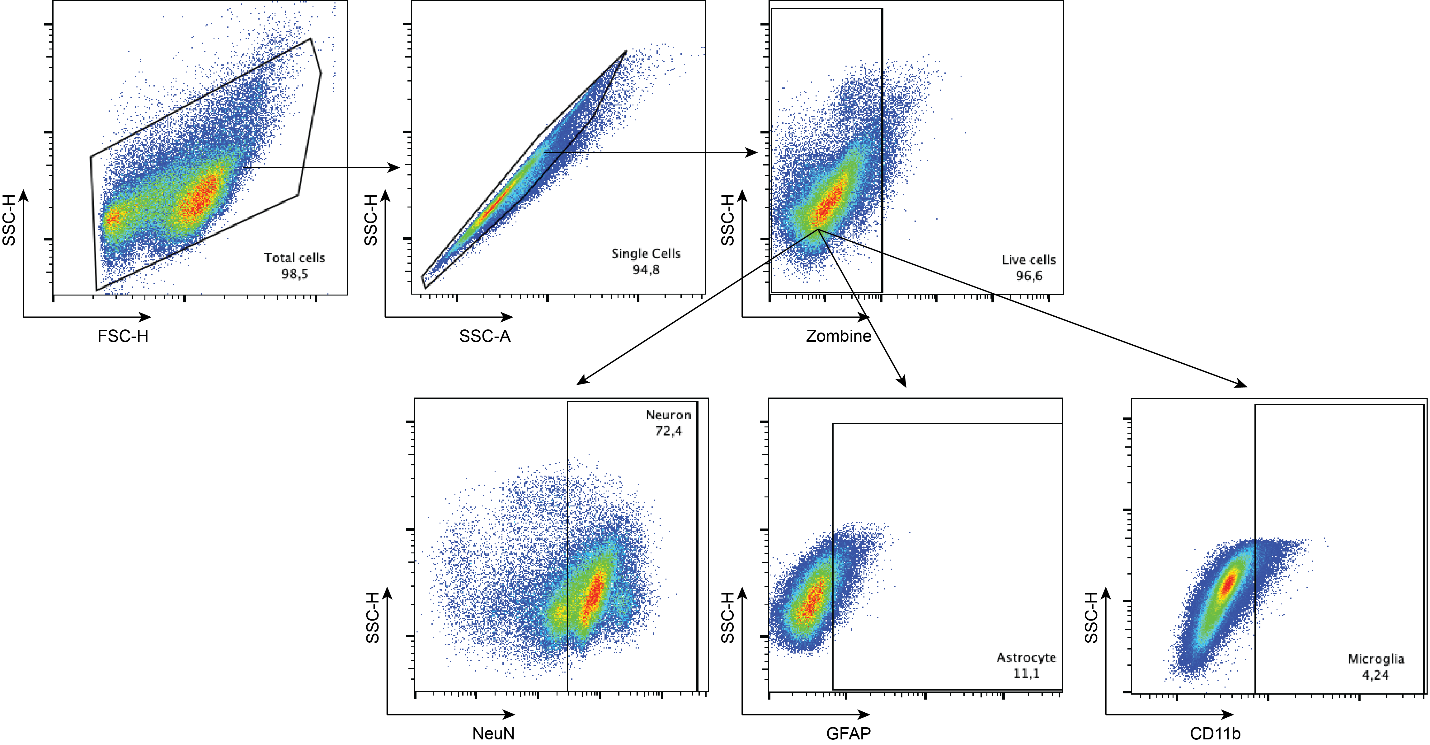


**Figure S19.** Flow gating strategy for analysis of P3B LNPs loading with Cre mRNA delivery to brain cells.


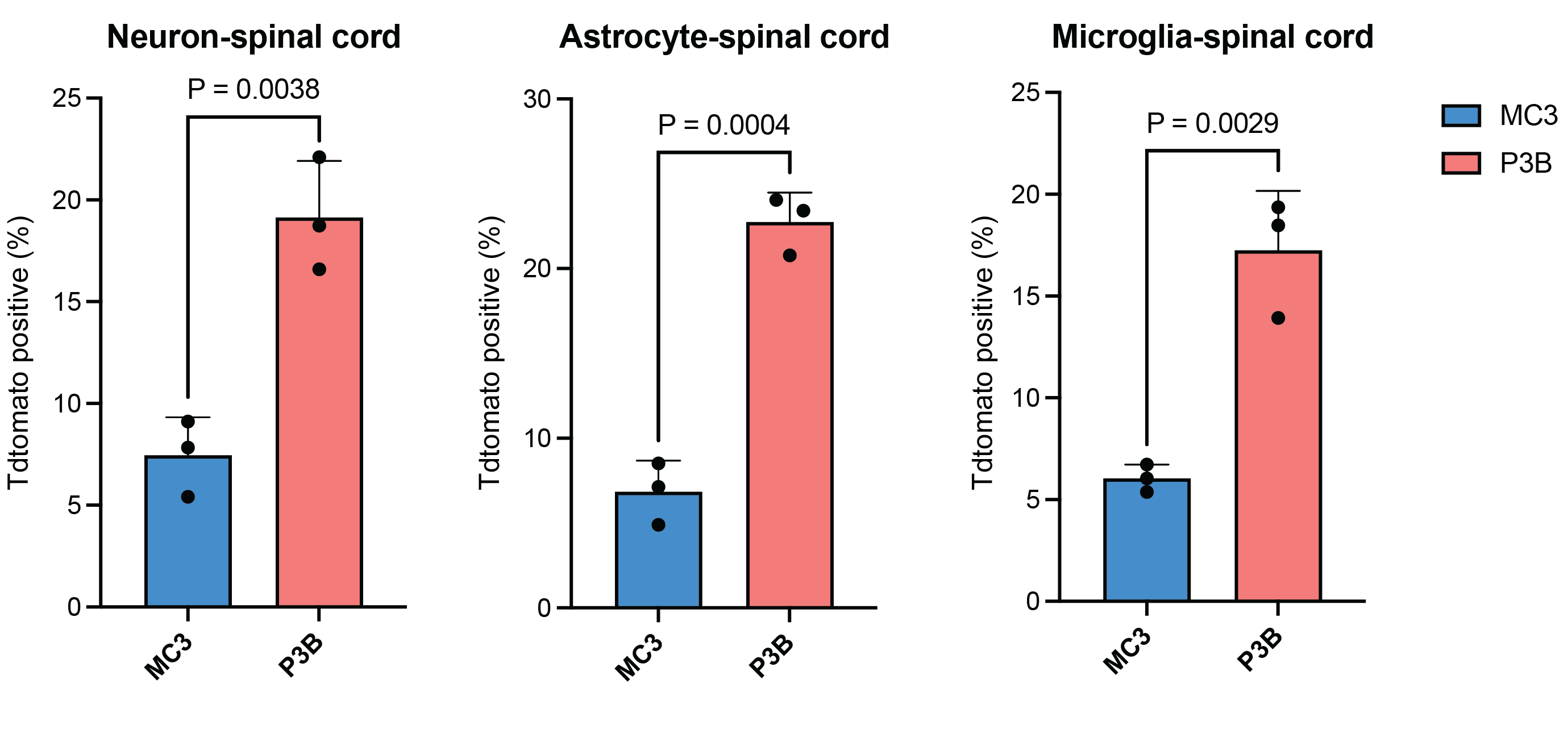


**Figure S20.** Quantification of tdTomato-positive neurons, astrocytes, and microglia across the spinal cord. Two-tailed unpaired Student’s t-tests were used to determine significance. Data are presented as mean ± s.d., n=3 biological replicates.


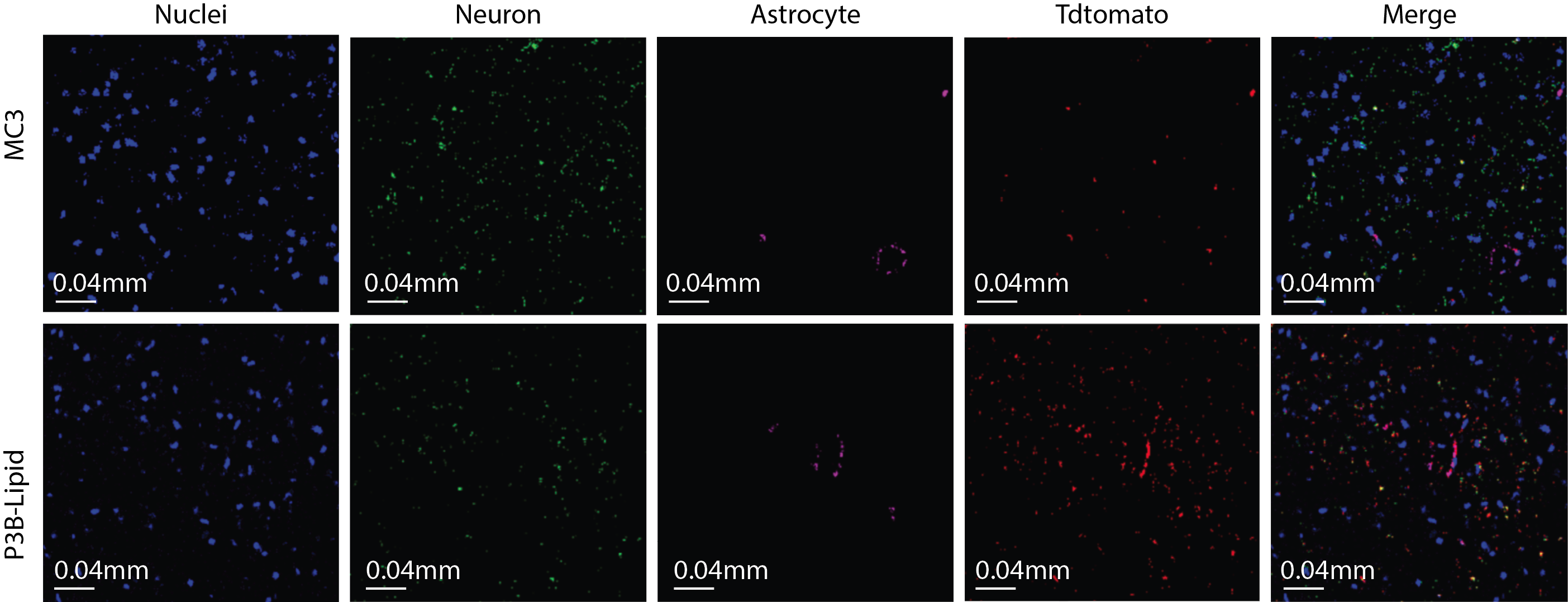


**Figure S21.** Representative immunofluorescence images showing tdTomato expression (red) following intrathecal administration of Cas9 mRNA/sgTOM LNPs formulated with MC3 (top) or P3B (bottom) in Ai9 reporter mice. Neurons were labeled with anti-NeuN (green) and astrocytes with anti-GFAP (magenta); nuclei were counterstained with DAPI (blue).


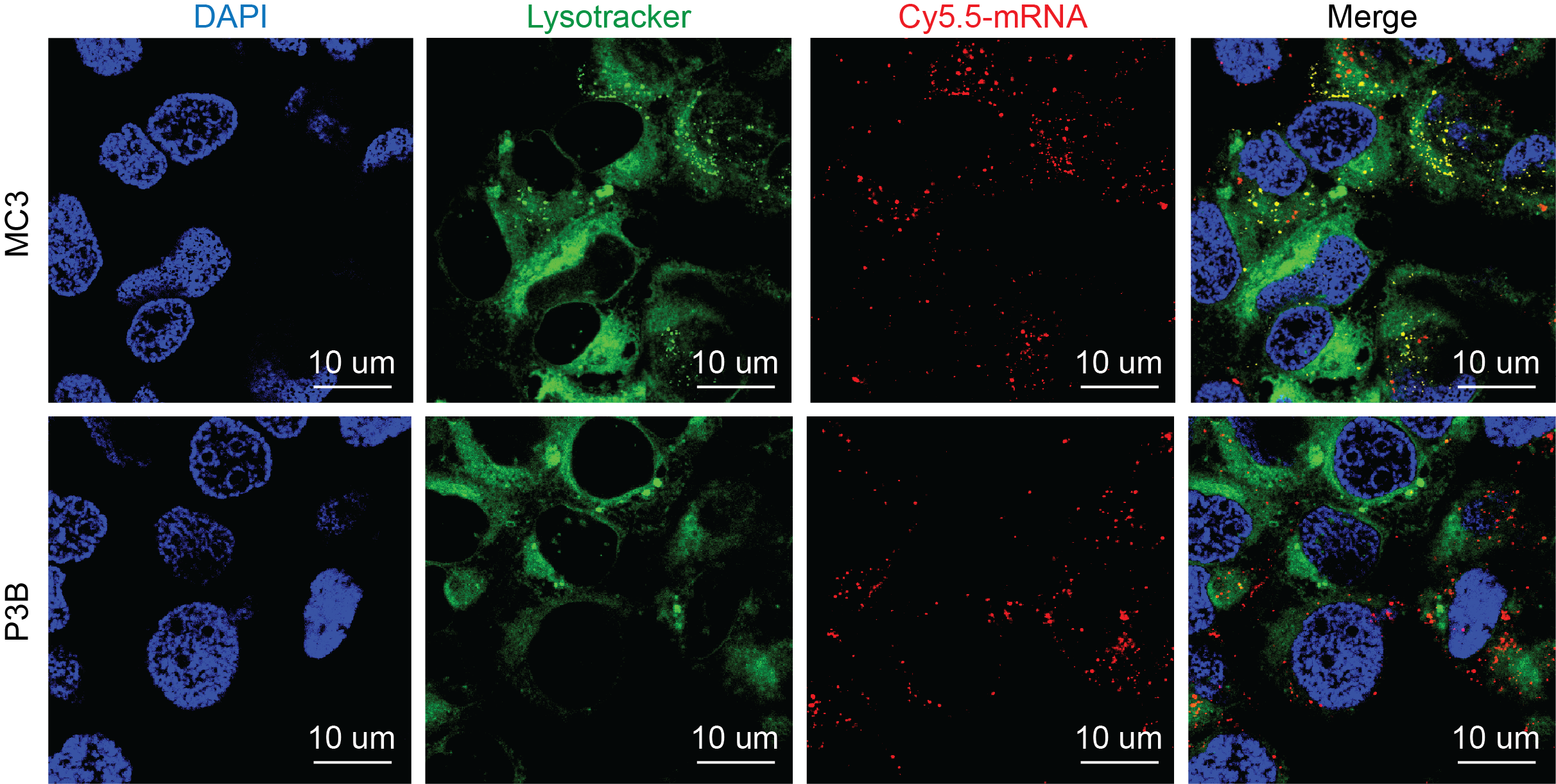


**Figure S22**. Representative images of cells treated with MC3-LNPs or P3B-LNPs encapsulating Cy5.5-labeled mRNA (red). Lysosomes were stained with Lysotracker (green), and nuclei were counterstained with DAPI (blue).


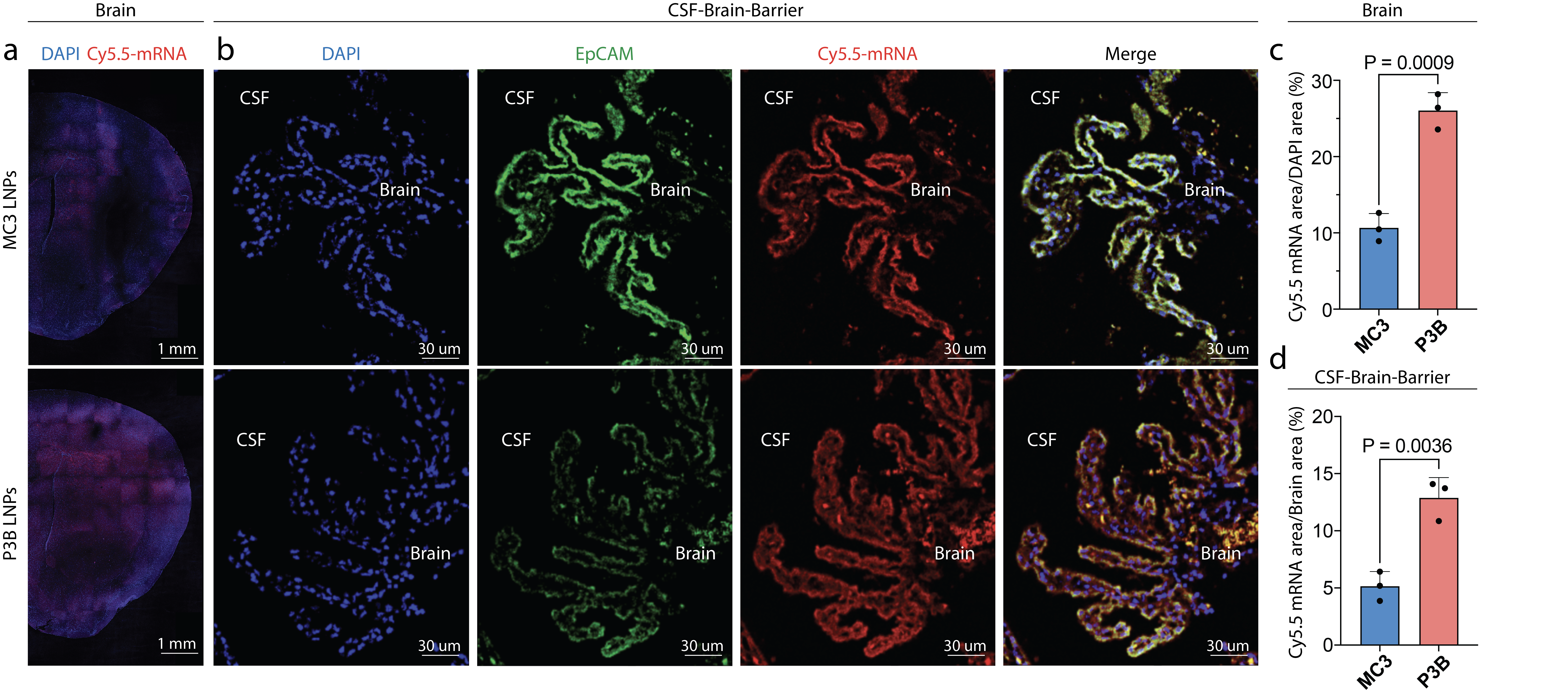


**Figure S23.** P3B-LNPs enable enhanced mRNA delivery across the CSF-brain barrier compared with MC3-LNPs. (a) Representative brain sections showing the distribution of Cy5.5-labeled mRNA (red) following intrathecal administration of MC3- or P3B-LNPs. Nuclei are counterstained with DAPI (blue); (b) High-magnification confocal images of the choroid plexus region illustrating mRNA localization at the CSF-brain interface. Immunostaining for EpCAM (green) delineates the epithelial layer of the CSF-brain barrier, while Cy5.5-mRNA (red) signals indicate LNP-delivered mRNA. (c-d) Quantitative analysis of Cy5.5-mRNA fluorescence normalized to DAPI-stained area in the brain (c) and at the CSF-brain barrier (d). Data represent mean ± s.d. from n = 3 biologically independent samples.


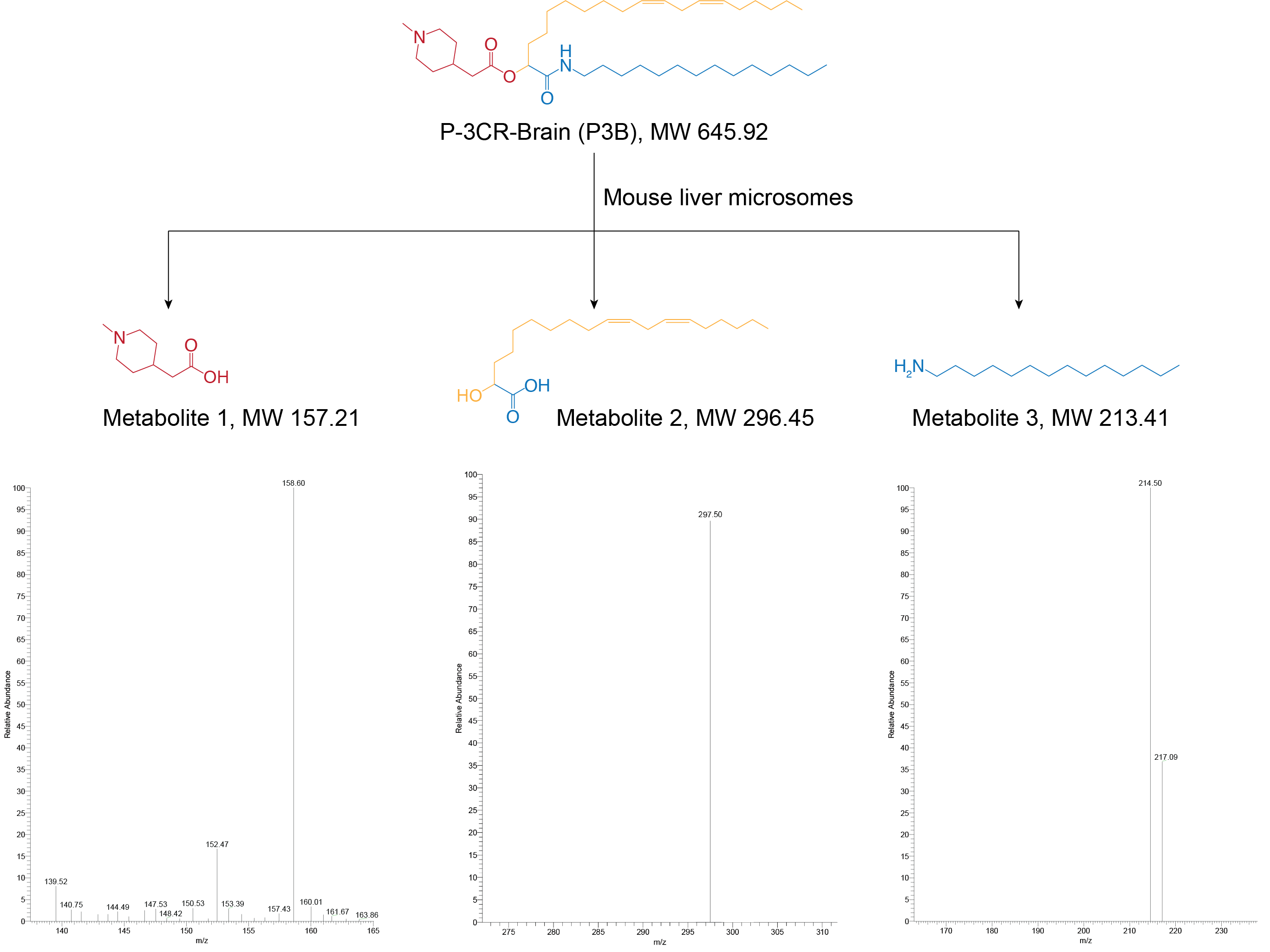


**Figure S24.** Metabolic profiling of P-3CR-Brain (P3B) in mouse liver microsomes. *In vitro* microsomal stability assays identified three major metabolites of P3B (MW = 645.92). LC-MS analysis revealed the formation of (i) Metabolite 1 (MW 157.21), corresponding to the hydrolyzed headgroup fragment; (ii) Metabolite 2 (MW 296.45), derived from partial cleavage of the ester linkage and oxidation of the intermediate; and (iii) Metabolite 3 (MW 213.41), representing the liberated alkyl amine tail.

**Table 1.** Details of formulation optimization presented in Figure 2.

| Formulation | Ionizable lipid/RNA wt. ratio | Ionizable lipid content (mol%) | Helper lipid type | Helper lipid content (mol%) | PEG content (mol%) |
| --- | --- | --- | --- | --- | --- |
| DOE 01 | 7.5 | 30 | DSPC | 10 | 0.5 |
| DOE 02 | 7.5 | 45 | DSPC | 20 | 1.25 |
| DOE 03 | 10 | 60 | DOPE | 30 | 0.5 |
| DOE 04 | 10 | 30 | DOPE | 20 | 2 |
| DOE 05 | 10 | 60 | DOPE | 10 | 0.5 |
| DOE 06 | 5 | 60 | DSPC | 30 | 1.25 |
| DOE 07 | 5 | 30 | DOPE | 30 | 0.5 |
| DOE 08 | 10 | 45 | DSPC | 30 | 2 |
| DOE 09 | 5 | 30 | DSPC | 30 | 2 |
| DOE 10 | 5 | 60 | DSPC | 20 | 0.5 |
| DOE 11 | 7.5 | 60 | DOPE | 30 | 2 |
| DOE 12 | 7.5 | 45 | DOPE | 20 | 1.25 |
| DOE 13 | 10 | 30 | DOPE | 10 | 1.25 |
| DOE 14 | 5 | 30 | DSPC | 10 | 2 |
| DOE 15 | 10 | 60 | DSPC | 10 | 2 |
| DOE 16 | 5 | 45 | DOPE | 10 | 0.5 |
| DOE 17 | 10 | 30 | DSPC | 30 | 0.5 |
| DOE 18 | 5 | 60 | DOPE | 10 | 2 |

**Table 2.** The sequence of sgRNA used in this paper.

| Formulation | Ionizable lipid/RNA wt. ratio | Ionizable lipid content (mol%) | Helper lipid type | Helper lipid content (mol%) | PEG content (mol%) |
| --- | --- | --- | --- | --- | --- |
| DOE 01 | 7.5 | 30 | DSPC | 10 | 0.5 |
| DOE 02 | 7.5 | 45 | DSPC | 20 | 1.25 |
| DOE 03 | 10 | 60 | DOPE | 30 | 0.5 |
| DOE 04 | 10 | 30 | DOPE | 20 | 2 |
| DOE 05 | 10 | 60 | DOPE | 10 | 0.5 |
| DOE 06 | 5 | 60 | DSPC | 30 | 1.25 |
| DOE 07 | 5 | 30 | DOPE | 30 | 0.5 |
| DOE 08 | 10 | 45 | DSPC | 30 | 2 |
| DOE 09 | 5 | 30 | DSPC | 30 | 2 |
| DOE 10 | 5 | 60 | DSPC | 20 | 0.5 |
| DOE 11 | 7.5 | 60 | DOPE | 30 | 2 |
| DOE 12 | 7.5 | 45 | DOPE | 20 | 1.25 |
| DOE 13 | 10 | 30 | DOPE | 10 | 1.25 |
| DOE 14 | 5 | 30 | DSPC | 10 | 2 |
| DOE 15 | 10 | 60 | DSPC | 10 | 2 |
| DOE 16 | 5 | 45 | DOPE | 10 | 0.5 |
| DOE 17 | 10 | 30 | DSPC | 30 | 0.5 |
| DOE 18 | 5 | 60 | DOPE | 10 | 2 |

| sgRNA | Target Sequences (5’ to 3’) | PAM |
| --- | --- | --- |
| sgTOM | AAGTAAAACCTCTACAAATG | TGG |
| sgLumA | GCCTCATTGATTAACGCCCA | GGG |

**Table 3.** Intrathecal injection procedure in mice.

| Procedure Stage | Experimental Details |
| --- | --- |
| Materials & Equipment | • Anesthetized mouse (typically with isoflurane)  • Sterile 30G needle with Hamilton syringe (10 µL)  • Antiseptic (e.g., 70% ethanol or iodine)  • Sterile gloves  • Heating pad (to maintain body temperature)  • Surgical drape or sterile workspace  • Optional: analgesics (per protocol)  • Personal protective equipment (PPE)  • 15-mL conical tube |
| Animal Preparation | 1. Anesthetize the mouse following approved protocols.  Animals will be anesthetized with isoflurane. The induction is carried out with 4-5%, and maintenance with 1.5% by continuous administration through a nose cone. The mice will be sufficiently sedated by gently pinching hind paws. If properly sedated, the mouse should not respond to the pinch. The eyes will be covered with eye lubricant using a sterile cotton tipped applicator  2. Place in prone position (belly down), with the head slightly lower than the hindquarters to expose the lower back.  3. Using an electric shaver, the hair around the lower spine will be shaved, starting from the base of the tail and spanning an area approximately 3 - 3 cm.  4. Apply hair remover cram on the shaved area. Leave the cream for max 30 seconds. Wipe down the cream with a dry gauze and then with sterile warm water.  5. Clean the injection site (between the L5–L6 vertebrae) with antiseptic solution. The area will be cleaned with an antiseptic 5–10% povidoneiodine solution, then wiped with 70% ethanol. |
| Injection Technique | 6. A 15-mL conical tube will be placed under the abdomen of the mouse to expose a bigger area of the interspinous ligament, which is punctured by the needle to access the intradural space.  7. This will also make it easier to find and grasp the iliac crest. The iliac crest will be identified by finding the two pits formed at the interface of the muscle and the hip bone. The injection site (L5–L6) will be directly above the iliac crest.  8. Identify the injection site: Locate the L5–L6 intervertebral space by palpation. The iliac crest will be identified by finding the two pits formed at the interface of the muscle and the hip bone. The injection site (L5–L6) will be directly above the iliac crest.  9. The position of the L6 vertebra will be determined by identifying the most protruding spinal process. Injection at this position reduces the possibility of spinal damage since this site is where the spinal cord ends and the cauda equina begins.  10. Stabilize the animal to minimize movement.  11. Insert the needle: The Hamilton 25-μL Syringe attached to a 30 G 0.5 in needle will be used to load with a test compound (5–10 μL)  12. Carefully insert the needle between the groove of L5 and L6 vertebrae and observe for a tail flick as this sign indicates a successful entry of the needle in the intradural space. Insert slowly between the vertebrae until you feel a “pop” or slight tail flick (indicating penetration into the intrathecal space).  Tip: Using fingernail, one should be able to locate the groove as well.7. Once tail flick is observed, immediately, but carefully, secure the needle position with one hand and inject the desired volume of substance with the other hand slowly. Inject the solution slowly (≤ 10 µL).  Tip: a volume between 5-10 μl is optimal as volume less than 5 μl is unreliable and a volume bigger than 10 μl leads to too much pressure.  13. Once injection is performed, move the mouse back to the cage to recover from anesthesia.  14. Repeat this injection at least 1 more time.  15. Withdraw the needle gently and apply slight pressure if there is any bleeding. |
| Post-Injection Monitoring | • Place the mouse on a heating pad and monitor until fully recovered.  • Watch for signs of distress, neurological issues, or complications.  • Record the procedure details per institutional protocol. |
| Critical Notes | • Accuracy is essential: incorrect technique can cause spinal damage or ineffective delivery.  • Practice on euthanized animals or under supervision is recommended before independent procedures.  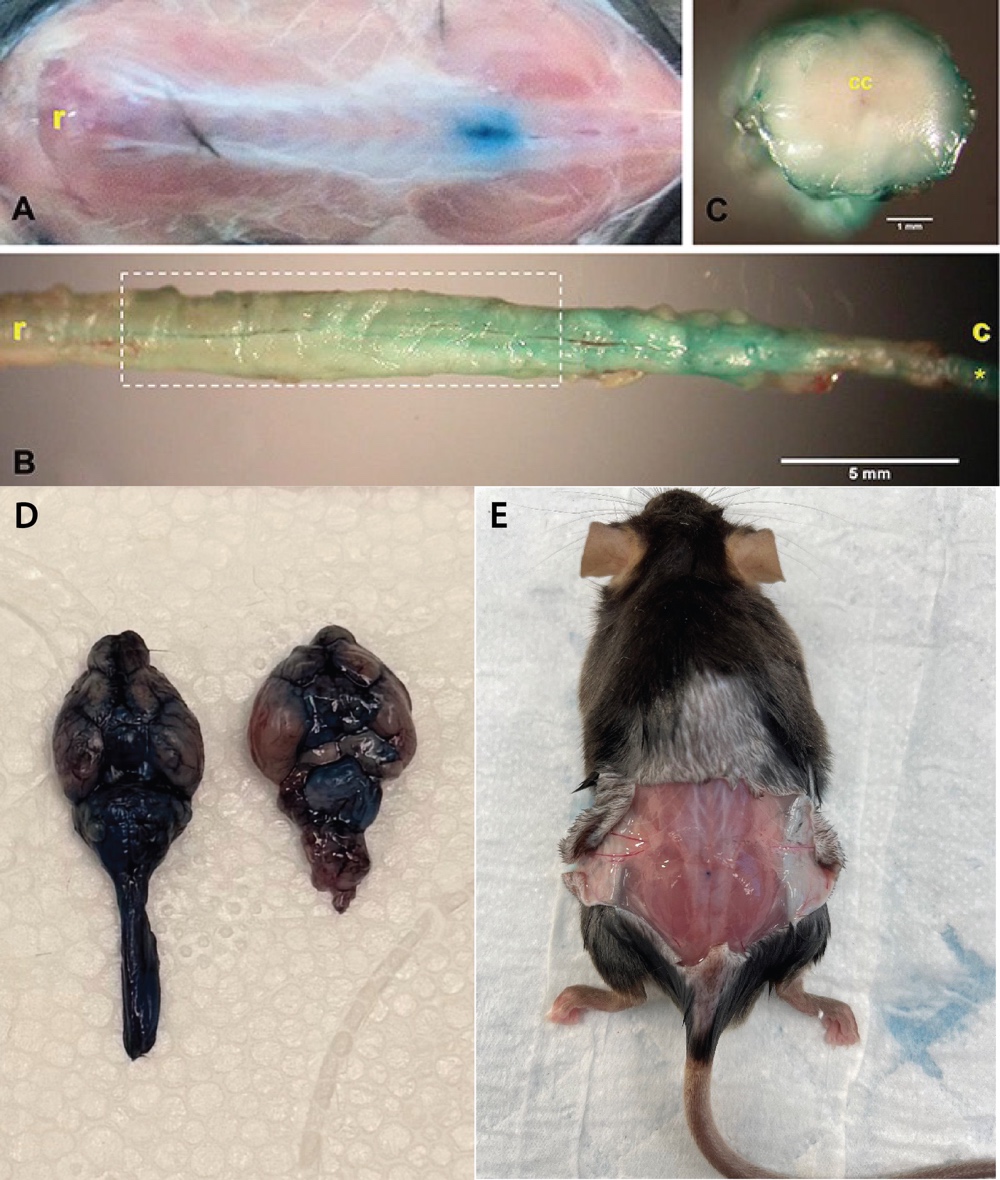  **Spinal cord dissected under a microscope following non-invasive, acute intrathecal injection with Fast Green dye.**  **A,** No visible tissue damage at the injection site, indicated by dye puncta; **B,** Excised spinal cord a few minutes post-injection showing gradual rostral diffusion of the dye. The white box highlights the lumbar region, and the star denotes the injection site; **C,** Dye staining confined to the surface of the spinal cord (L3-L5 segments) without penetration into the parenchyma; r = rostral, c = caudal, cc = central canal; **D,** Specific staining observed in the brain; **E,** Close-up view of the intrathecal injection puncture site. |

# Section D. References

[1] Y.-X. Si, P.-F. Zhu, S.-L. Zhang, Synthesis of Isocyanides by Reacting Primary Amines with Difluorocarbene, Organic Letters, 22 (2020) 9086-9090.

[2] Y. Xu, F. Gong, A. Golubovic, A. Strilchuk, J. Chen, M. Zhou, S. Dong, B. Seto, B. Li, Rational design and modular synthesis of biodegradable ionizable lipids via the Passerini reaction for mRNA delivery, Proceedings of the National Academy of Sciences, 122 (2025) e2409572122.
